# An Overview of Chromic Transition Metal Oxide Thin Films

**DOI:** 10.3390/ma19142943

**Published:** 2026-07-08

**Authors:** Gheorghe Ghilețchii, Alexandru Varzari, Ştefan-Andrei Irimiciuc, Ján Lančok, Sergiu Vatavu

**Affiliations:** 1 Physics of Semiconductors and Devices Lab, Faculty of Physics and Engineering, Moldova State University, 2009 Chisinau, Moldova; ghiletchii.gheorghe@usm.md (G.G.); alexandru.varzari@usm.md (A.V.); sergiu.vatavu@usm.md (S.V.); 2 Institute of Physics of the Czech Academy of Science, 182 00 Prague, Czech Republic; lancok@fzu.cz; 3 National Institute for Laser, Plasma and Radiation Physics, 077125 Magurele, Romania

**Keywords:** chromism, thermochromism, electrochromism, transition metal oxides, electrochromic/thermochromic smart windows

## Abstract

Transition metal oxides constitute an important materials platform for chromic phenomena because their optical response is strongly coupled to the changes in electronic structure, phase state, carrier concentration, and defect chemistry. This review discusses selected transition metal oxide thin films, with emphasis on VO_2_ and other vanadium oxides, WO_3_, NiO, and TiO_2_. The review summarizes the structural and electronic characteristics of these representative oxide systems and highlights the role of phase composition, crystal structure, oxygen non-stoichiometry, and defect chemistry in determining their optical response. The main thin film preparation routes, including pulsed laser deposition, magnetron sputtering, sol–gel and aerosol spray methods, atomic layer deposition, chemical vapor deposition, electrochemical routes, and molecular beam epitaxy, are reviewed with respect their influence on obtained thin films. Particular attention is given to applications in thermochromic VO_2_-and electrochromic WO_3_/NiO-based smart windows, and transition metal oxide-based gasochromic hydrogen sensors. Key challenges related to transition temperature tuning, luminous transmittance, solar modulation, optical contrast, cycling stability, ion transport and large-area integration are also discussed. Overall this review provides a comparative overview of selected transition metal oxide thin films by connecting material chemistry and physics, thin film preparation technology and functionality.

## 1. Introduction

Smart materials are materials capable of undergoing reversible and controllable changes in their physical or chemical properties under external stimuli such as temperature, light, electric field, mechanical stress, gas atmosphere, or chemical environment [1,2,3]. Unlike conventional materials, which are usually regarded as passive static systems with fixed functional parameters, smart materials behave as dynamic systems whose response can adapt to changing operating conditions. Their ability to modulate electrical, optical, magnetic, mechanical, or thermal properties makes them relevant to the transition from static material platforms toward responsive and adaptive systems.

Within this broader group, materials with adaptive optical response form a particularly important subgroup, since external stimuli can directly modify optical transmittance, reflectance, absorption, apparent color, or thermal emissivity. Such control over light–matter interaction is especially valuable because optical response is immediately linked to visual perception, daylight and solar energy management, thermal regulation, and signal readout. From a technological point of view, these changes can be readily monitored visually or spectroscopically, which facilitates their use in coatings, smart windows (SWs), sensors, and other optoelectronic devices [4,5,6,7,8]. Among these materials, chromic systems are of particular interest because they reversibly modulate the spectral response and/or visible appearance under external stimuli.

Chromic materials are actively investigated for smart and energy-efficient windows, building-integrated thermal-management coatings, optical and gas-sensing systems, visual indicators, adaptive camouflage, information-storage and display-related concepts, and thin film optoelectronic devices [5,8,9,10,11].

**Figure 1:**
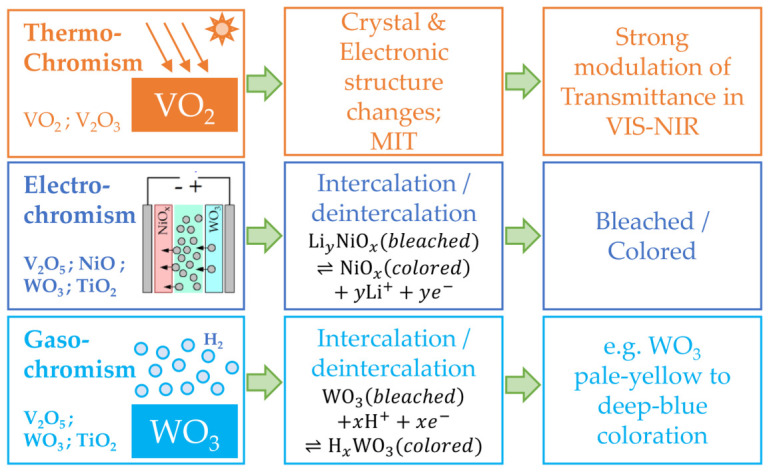
Chromism mechanism in selected TMOs.

Transition metal oxides (TMOs) constitute a broad class of inorganic materials with a remarkable diversity of crystal structures and of structural, electronic, and optical properties [12]. Depending on composition, bonding geometry, and correlation strength, TMOs can behave as both band insulators (or semiconductors) and correlated metal. In addition, many TMOs exhibit metal-insulator (MIT) phase transitions, magnetism, multiferroicity, and other emergent phenomena [13]. Owing to this versatility, TMOs have become an important functional platform for electronics, optoelectronics, sensing, and energy-related technologies [14,15,16]. For chromic functionality, TMOs are especially attractive because their optical response is strongly coupled to their electronic and structural state [13]. Changes in carrier concentration, oxidation state, defect chemistry, and phase state appreciably transform the electronic structure and thereby modify optical properties [13,17]. As a result, external stimuli can induce pronounced and often reversible optical changes, making TMOs a particularly favorable platform for chromism [6,8] (Figure 1).

Although chromic behavior can also be investigated in bulk materials, thin films are the most relevant form for practical optical applications. Film geometry offers direct control over thickness, composition and stoichiometry, crystallinity, surface and interface structure, and defect concentration through deposition and post-treatment conditions, while also enabling multilayer architectures and large-area coatings [18]. For this reason, thin film TMOs dominate chromism applications. Accordingly, the emphasis of the present review is placed on selected chromic TMO thin films. The literature on individual chromic TMOs has expanded rapidly, whereas discussions of electronic/structural properties, preparation routes, and device integration often remain fragmented across separate material systems. This creates a clear need for a review that connects these subjects.

Accordingly, the present review aims to: summarize the key structural, electronic, and optical features of representative chromic TMOs; review the principal technological routes used to obtain selected TMO thin films and highlight the main application areas of chromic oxide thin films.

At the same time, this review does not seek to provide an exhaustive survey of all oxide materials, synthesis approaches, or device architectures. Rather, its focus is placed on chromism-related structural, electronic, and optical aspects of selected TMO systems. The material scope of this review is focused on selected TMOs thin films, namely VO_2_ and other vanadium oxides, WO_3_, NiO, and TiO_2_. These materials were chosen as representative systems because they are among the most intensively studied and most frequently discussed chromic TMOs in the literature. In addition, they reflect distinct classes of chromic behavior and different physical routes to optical modulation, thereby providing a sufficiently broad yet coherent basis for comparative discussion. The selected oxides therefore do not represent an exhaustive list of chromic TMOs, but rather a set of benchmark material platforms through which the principal features of chromism in oxide thin films can be analyzed.

**Figure 2:**
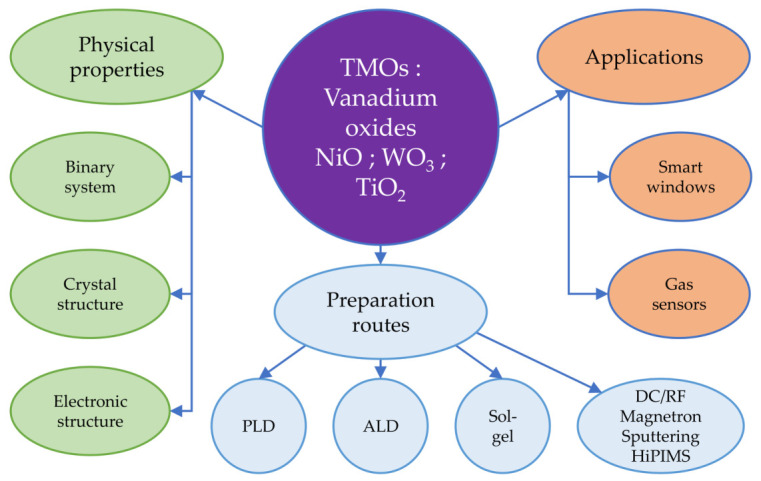
Article map.

The article is organized into several major sections (Figure 2), each corresponding to a different level of analysis: the material platform section examines the structural and electronic proprieties of the selected TMOs; the technology section reviews the main preparation routes for TMO thin films; the applications section outlines the principal directions in which chromic properties of TMO thin films are utilized.

## 2. Materials Platform: Structural and Electronic Properties of Selected TMOs

### 2.1. Vanadium Oxides

#### 2.1.1. V–O Binary Phase Diagram

The V–O phase diagram [19] reflects the thermodynamic equilibrium of phases over a wide temperature range and illustrates the complex phase relations that arise from the ability of vanadium to form oxides with different stoichiometries, oxidation states, and structural modifications. Despite the large number of phases shown in the diagram, the number of thermodynamically stable stoichiometric compounds is limited, whereas a significant fraction of the observed phases corresponds to solid-solution regions or metastable states. A characteristic feature of the V–O phase diagram is the presence of extended non-stoichiometric regions in both the V-rich and O-rich parts of the diagram. This behavior demonstrates the important role of oxygen content as a controlling parameter for phase stability in the V–O system.

In the V-rich region of the V–O phase diagram, four oxygen-containing solid solutions can be distinguished: α-, β-, γ-, and δ-V phases [20]. In the oxide region, five stoichiometric vanadium oxides are thermodynamically stable: V_2_O_3_ with a corundum-type trigonal structure ($R\bar{3}c$, No. 167), V_3_O_5_ with a monoclinic structure (P2/c, No. 13), high-temperature rutile-type VO_2_ with a tetragonal structure ($P4_{2}/mnm$, No. 136), V_3_$O_{7}$ with a monoclinic structure (C2/c, No. 15), and V_2_O_5_ with an orthorhombic structure (Pmmn, No. 59).

In addition to these stable stoichiometric compounds, the V–O system contains metastable and non-stoichiometric oxides that are typically formed under non-equilibrium, oxygen-deficient, or oxygen-rich conditions. Oxygen-deficient compositions are associated with mixed-valence Magnéli phases compositionally located between V_2_O_3_ and VO_2_, whereas oxygen-rich compositions are associated with Wadsley phases located between VO_2_ and V_2_O_5_. These homologous series describe how the V–O system accommodates oxygen deficiency or excess and can be represented as formal combinations of neighboring stable oxides.

The Magnéli phases are mixed-valence systems in which V^3+^ and V^4+^ ions coexist, and the average oxidation state of vanadium increases with increasing *n*. Their key structural feature is the presence of crystallographic shear planes, which arise from ordered shear transformations of the rutile-like VO_6_ octahedral framework. The resulting disruptions of the VO_6_ octahedral chains are accommodated by collective shifts of the octahedra, forming periodic shear planes whose spacing is determined by the parameter *n*:

(1) $$V_{n}O_{2n-1}=V_{2}O_{3}+\left(n-2\right){\mathrm{VO}}_{2},\;3\leq n\leq 9.$$

The Wadsley phases occupy the oxygen-rich side of the V–O phase diagram and can be represented as linear combinations of VO_2_ and V_2_O_5_:

(2) $$V_{n}O_{2n+1}=V_{2}O_{5}+\left(n-2\right){\mathrm{VO}}_{2},\;n\geq 3.$$

The Wadsley phases are likewise mixed-valence systems involving V^4+^ and V^5+^ ions. Unlike Magnéli phases, in which oxygen deficiency is accommodated by crystallographic shear planes, excess oxygen in Wadsley phases is accommodated through rearrangements of the octahedral framework and the formation of more complex layered or block-like structures. Their periodicity and connectivity are governed by the structural parameter *n*.

#### 2.1.2. Crystal Structure of Vanadium Dioxide VO_2_

Below the MIT temperature, $T_{c}\approx 68\;{}^{\circ}C$, VO_2_ exhibits insulating behavior and adopts a monoclinic structure. The M1 phase is the thermodynamically stable low-temperature phase of stoichiometric VO_2_, whereas the M2 phase is metastable and can be stabilized by applied stress, chemical doping, or epitaxial strain.

The M1 phase has the monoclinic space group $P2_{1}/c$ (No. 14) and represents the stable low-temperature stoichiometric phase under ambient pressure. Its lattice parameters are $a_{M1}=5.751\;Å$, $b_{M1}=4.537\;Å$, $c_{M1}=5.382\;Å$, $\alpha =\gamma =90^{\circ }$, and $\beta_{M1}=122.64^{\circ }$ [21].

Structurally, the M1 phase is characterized by complete V–V dimerization along chains derived from the rutile $c_{R}$ direction. Each vanadium atom participates in the formation of V–V dimers, leading to a doubling of the unit cell and a lowering of the lattice symmetry relative to the high-temperature rutile phase. In addition to dimerization, the vanadium atoms undergo lateral displacements accompanied by rotations of the surrounding VO_6_ octahedra. These distortions break the equivalence of oxygen sites and result in two crystallographically distinct oxygen positions. The M1 structure therefore reflects a cooperative lattice distortion involving both V–V pairing and octahedral tilting, which plays a central role in stabilizing the insulating state of VO_2_ below $T_{c}$.

The M2 phase is a metastable monoclinic phase with the space group C2/m (No. 12). Its lattice parameters are $a_{M2}=9.066\;Å$, $b_{M2}=5.797\;Å$, $c_{M2}=4.525\;Å$, $\alpha =\gamma =90^{\circ }$, and $\beta_{M2}=91.88^{\circ }$ [22].

Unlike the M1 phase, the M2 phase is not generally observed in bulk stoichiometric VO_2_ under ambient conditions but can be stabilized by chemical doping, applied stress, or epitaxial strain [22,23]. A defining feature of the M2 phase is the inequivalent behavior of vanadium chains. In this structure, only half of the vanadium chains exhibit V–V dimerization, while the remaining chains retain a non-dimerized configuration.

**Figure 3:**
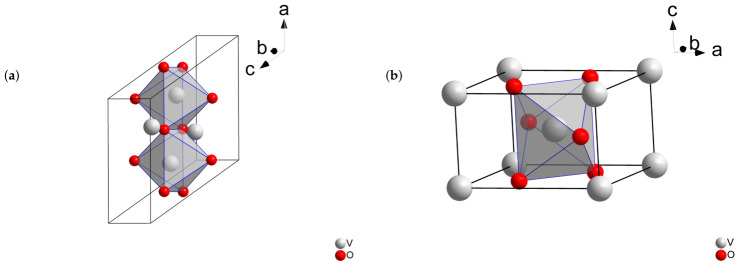
Crystal structure of (**a**) M1 and (**b**) rutile VO_2_.

In the M1 phase, vanadium atoms and the two crystallographically distinct oxygen atoms occupy the Wyckoff position 4e. The corresponding symmetry-equivalent positions are given by ±(x;y;z) and ±(x;1/2−y;1/2+z). The fractional atomic coordinates are summarized in Table 1. Based on these atomic parameters, the crystal structure of VO_2_ is shown in Figure 3a.

All crystal structure figures were generated using Diamond 4 software, based on crystallographic data reported in the corresponding cited references.

Above $T_{c}$, VO_2_ transforms into a metallic phase with the rutile crystal structure. This high-temperature phase crystallizes in the tetragonal space group $P4_{2}/mnm$ (No. 136). Its lattice parameters are $a_{R}=b_{R}=4.556\;Å$, $c_{R}=2.860\;Å$, and $\alpha =\beta =\gamma =90^{\circ }$ [22] (Table 2, Figure 3b).

Across the MIT at $T_{c}$, neighboring vanadium atoms within each chain shift toward one another, leading to V–V pairing, while the surrounding VO_6_ octahedra undergo accompanying rotations. This cooperative distortion produces a quasi-one-dimensional Peierls-like instability with contributions from electron correlations. It breaks the uniformity of the V chains, creates two distinct oxygen sites, and lowers the lattice symmetry from the rutile R phase to the monoclinic M1 phase.

**Figure 4:**
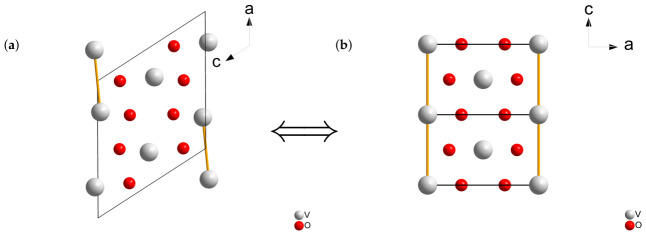
Structural change from (**a**) M1 along $a_{M1}$ to (**b**) rutile along $c_{R}$.

The V–V distances become alternating, with a dimer bond length of ∼2.62Å and an interdimer separation of ∼3.12Å. Upon heating, this distortion is reversed and the structure transforms back into the rutile phase [24] (Figure 4); this process is reversible.

#### 2.1.3. Crystal Structure of Divanadium Trioxide V_2_O_3_

**Figure 5:**
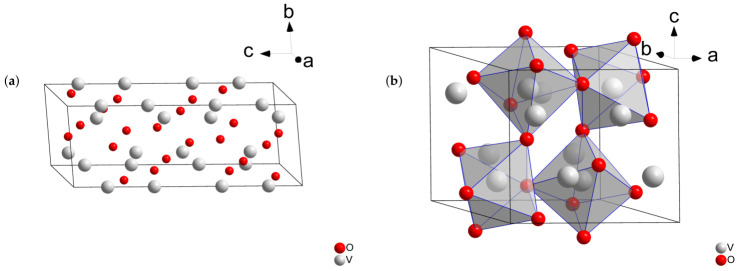
Crystal structures of $V_{2}O_{3}$ (**a**) corundum and (**b**) monoclinic.

V_2_O_3_ is a strongly correlated TMO that exhibits an MIT accompanied by a structural phase transformation at $T_{c}\approx 155\;K$. At higher temperatures, it adopts a corundum-type structure $R\bar{3}c$ (No. 167) with lattice parameters a=b=4.951Å, c=14.003Å, $\alpha =\beta =90^{\circ }$, and $\gamma =120^{\circ }$ [25] (Table 3, Figure 5a).

In this phase, all vanadium atoms are crystallographically equivalent and are octahedrally coordinated by oxygen, forming a three-dimensional lattice of edge-sharing VO_6_ octahedra. This high-symmetry structure corresponds to the metallic state of V_2_O_3_.

Upon cooling below $T_{c}$, V_2_O_3_ undergoes a structural transformation into a monoclinic insulating phase with space group I2/a (No. 15) and lattice parameters a=7.274Å, b=5.005Å, c=5.551Å, $\alpha =\gamma =90^{\circ }$, and $\beta =96.78^{\circ }$ [26] (Table 4, Figure 5b). The monoclinic distortion lifts the equivalence of the vanadium sites and introduces subtle rearrangements of the VO_6_ octahedra, which are closely associated with the onset of electronic localization in the insulating state.

#### 2.1.4. Crystal Structure of Divanadium Pentoxide V_2_O_5_

V_2_O_5_ represents the most oxygen-rich compound and the highest oxidation state phase in the V–O system, with vanadium in the formal V^5+^ ($d^{0}$) electronic configuration. In contrast to VO_2_ and V_2_O_3_, V_2_O_5_ does not exhibit an MIT and remains insulating over a wide temperature range.

**Figure 6:**
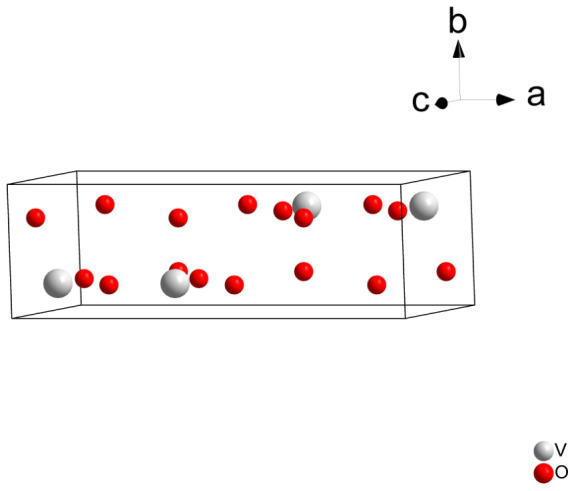
Crystal structures of V_2_O_5_.

V_2_O_5_ crystallizes in an orthorhombic structure with space group Pmmn (No. 59) and lattice parameters a=11.512Å, b=3.564Å, c=4.368Å, $\alpha =\beta =\gamma =90^{\circ }$ [27] (Table 5, Figure 6). The crystal structure is highly anisotropic and consists of layers formed by distorted VO_5_ square pyramids sharing edges and corners.

#### 2.1.5. Electronic Structure of Vanadium Dioxide VO_2_

Vanadium dioxide (VO_2_) is a correlated oxide in which the electronic structure undergoes a pronounced reconstruction across the coupled structural–electronic MIT [28,29,30]. In VO_2_, vanadium is formally in the V^4+^ state with a $3d^{1}$ configuration; therefore, the electronic states near the Fermi level are governed primarily by V-3d states hybridized with O-2p states [24,31].

**Figure 7:**
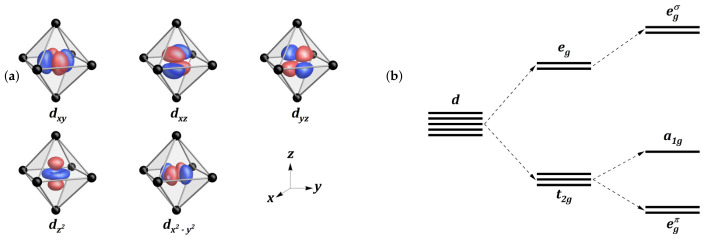
Schematic illustration of (**a**) the five d orbitals in an octahedral coordination and (**b**) the corresponding crystal-field splitting: $d\to t_{2g}+e_{g}$, followed by $t_{2g}\to a_{1g}+e_{g}^{\pi }$ and $e_{g}\to e_{g}^{\sigma }$.

In an approximately octahedral VO_6_ environment, the five V-3d orbitals are split by the crystal field into a low-energy $t_{2g}$ manifold ($d_{xy}$, $d_{xz}$, $d_{yz}$) and a high-energy $e_{g}$ manifold ($d_{z^{2}}$, $d_{x^{2}-y^{2}}$), with a characteristic splitting energy $\Delta_{O}$ (occasionally denoted 10Dq) (Figure 7a). Since the system hosts a single *d* electron, the $t_{2g}$ states primarily govern the electrical conductivity and the low-energy optical response. The actual local symmetry in VO_2_ deviates from the ideal $O_{h}$ symmetry; therefore, the $t_{2g}$ manifold further splits into sublevels: $a_{1g}$, a singlet often denoted $d_{\|}$, with the largest projection along the rutile axis $c_{R}$ and strong overlap along the V–V chains; and $e_{g}^{\pi }$, a doublet consisting of two orbital components with a smaller projection along $c_{R}$ and predominantly π-type overlap with O-2p states (*p*–*d*π hybridization) [24,31,32] (Figure 7b).

**Figure 8:**
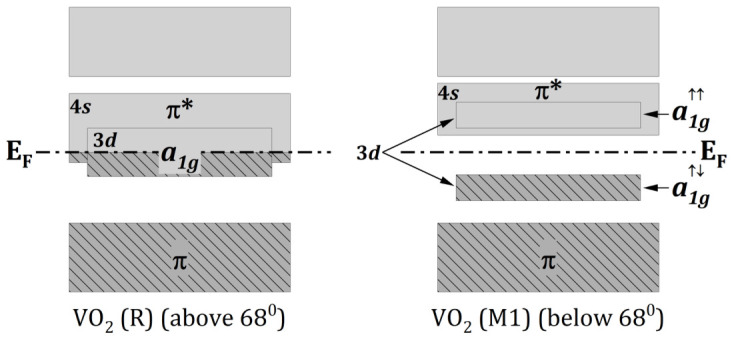
Schematic electronic structure of ${\mathrm{VO}}_{2}$.

In the high-temperature rutile phase, V atoms form quasi-linear chains along the $c_{R}$ axis. The $a_{1g}$ orbital exhibits the largest overlap along the V–V chains and gives rise to a relatively dispersive band, whereas the two $e_{g}^{\pi }$ components hybridize more strongly with the oxygen 2p states via π-channels. As a result, the $t_{2g}$ bands are partially filled, the Fermi level crosses the V–3d states, and the system exhibits metallic behavior. Importantly, the metallic state of ${\mathrm{VO}}_{2}$ is correlated: the effective masses and spectral characteristics of quasiparticles differ markedly from the predictions of a simple single-electron picture due to substantial Coulomb correlations [29,30] (Figure 8). Upon lowering the temperature, VO_2_ transforms into the monoclinic M1 phase, accompanied by two key lattice distortions: dimerization of V atoms along the chains and tilting of the VO_6_ octahedra. Dimerization splits the $a_{1g}$ band into bonding and antibonding components due to the enhanced V–V overlap along $c_{R}$. Simultaneously, octahedral tilting modifies the p−d hybridization and the energetic position of the $e_{g}^{\pi }$ states, typically shifting them upward relative to the Fermi level. As a result, the single *d*-electron occupies the lower $a_{1g}$ subband, while the upper $a_{1g}$ component and the $e_{g}^{\pi }$ states lie at higher energies, leading to the opening of a band gap and insulating behavior [24,29,31,32] (Figure 8).

#### 2.1.6. Electronic Structure of Divanadium Trioxide V_2_O_3_

Unlike VO_2_, where the MIT is strongly intertwined with a pronounced dimerization-driven band rearrangement, the MIT in V_2_O_3_ is commonly described as a Mott–Hubbard-type transition [33]. In stoichiometric V_2_O_3_, vanadium is formally in the V^3+^ state with a $3d^{2}$ configuration. Vanadium ions are coordinated by oxygen in an approximately octahedral VO_6_ environment, so the V-3d states are split by the crystal field into a low-energy $t_{2g}$ manifold and a high-energy $e_{g}$ manifold, separated by $\Delta_{O}$. Because the relevant *d* electrons primarily occupy the $t_{2g}$ region, the low-energy electronic structure near the Fermi level is dominated by $t_{2g}$-derived states hybridized with O-2p states.

In the corundum structure, the local symmetry deviates from ideal $O_{h}$ symmetry due to a trigonal distortion of the VO_6_ octahedra, which further splits the $t_{2g}$ manifold into an $a_{1g}$ singlet and an $e_{g}^{\pi }$ doublet. This trigonal splitting and the resulting orbital polarization ($a_{1g}$ vs. $e_{g}^{\pi }$) play an important role in shaping the low-energy spectrum and the MIT [34,35,36].

In the paramagnetic metallic (PM) regime, realized, for example, at elevated temperatures or under conditions that effectively increase the electronic bandwidth *W*, the $t_{2g}$-derived bands are partially filled and the Fermi level crosses V-3d states, yielding metallic transport. However, the metallic phase is strongly correlated: quasiparticle bands are substantially renormalized, with reduced bandwidth and enhanced effective masses, and the spectral function typically exhibits reduced coherent weight near $E_{F}$, accompanied by incoherent features at higher binding energies associated with Hubbard physics [37,38].

Upon entering an insulating regime, e.g., the antiferromagnetic insulating (AFI) phase at lower temperatures for stoichiometric material or a paramagnetic insulating state under specific tuning, the coherent spectral weight at the Fermi level is strongly suppressed and a gap opens. In the Mott–Hubbard representation, this corresponds to a redistribution of spectral weight from a coherent quasiparticle peak into lower and upper Hubbard bands, driven by the dominance of the on-site Coulomb repulsion *U* over the effective bandwidth *W*. The trigonal crystal-field splitting and orbital occupancy of the $a_{1g}$ and $e_{g}^{\pi }$ submanifolds can influence the detailed character of the transition, including the degree of orbital selectivity and the magnitude of the gap [33,36,37,38].

#### 2.1.7. Electronic Structure of Divanadium Pentoxide V_2_O_5_

Stoichiometric V_2_O_5_ is an insulating, charge-transfer (CT) TMO. Vanadium in V_2_O_5_ is formally in the V^5+^ oxidation state with a $3d^{0}$ electronic configuration, i.e., the V-3d shell is empty and there are no intrinsic carriers in a partially filled *d* band. The valence-band maximum is dominated by O-2p states, whereas the conduction-band minimum has predominantly V-3d character with substantial O-2p/V-3d hybridization; therefore, the Fermi level lies inside the band gap [39,40,41]. The stable ambient-pressure phase of V_2_O_5_ is orthorhombic and layered [27,42]. The V-3d states may still be approximately separated into $t_{2g}$-like and $e_{g}$-like groups. However, the low local symmetry and pronounced structural distortions substantially lift the degeneracy and modify the orbital energies compared with an ideal $O_{h}$ octahedron [39,42]. Unlike VO_2_ and V_2_O_3_, V_2_O_5_ is not characterized by a temperature-driven MIT under ambient conditions [43].

### 2.2. Nickel Oxide

#### 2.2.1. Ni–O Binary Phase Diagram

In the Ni–O system [44,45], nickel oxide NiO is the only oxide that remains thermodynamically stable over a wide range of temperatures and oxygen partial pressures, whereas other nickel oxides appear only under restricted conditions and are generally metastable, such as Ni_3_O_4_, Ni_3_O_3_ and NiO_2_ [46]. Although the phase diagram indicates a strict Ni:O ratio of 1:1, in practice NiO almost always exists as a nickel-deficient oxide of the form ${\mathrm{Ni}}_{1-x}$O ($x\approx 10^{-3}-10^{-1}$). Charge compensation in these non-stoichiometric states occurs through the formation of nickel vacancies, the presence of Ni^3+^ ions [47], and the emergence of localized hole polarons, which govern the p-type conductivity and significantly affect the optical properties of the material.

#### 2.2.2. Crystal Structure of Nickel Oxide

**Figure 9:**
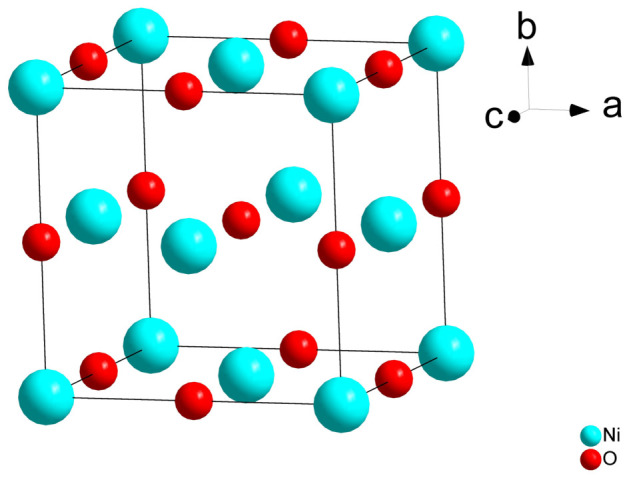
Crystal structure of NiO.

Stoichiometric NiO crystallizes in the rock-salt structure, belonging to the space group $Fm\bar{3}m$ (No. 225). The lattice parameter of the cubic unit cell is a=4.177Å [48] (Table 6, Figure 9). Each ${\mathrm{Ni}}^{2+}$ ion is octahedrally coordinated by six $O^{2-}$ ions, forming NiO_6_ octahedra, while each oxygen atom is similarly coordinated by six nickel ions.

#### 2.2.3. Electronic Structure of Nickel Oxide

**Figure 10:**
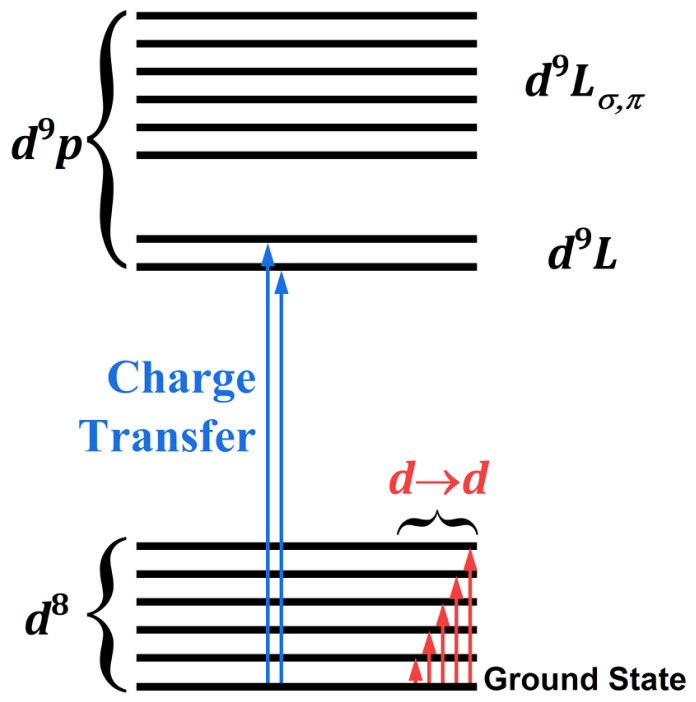
Schematic electronic diagram of ${\mathrm{NiO}}_{6}^{-10}$ cluster illustrating intra-atomic d→d multiplet excitations within the $d^{8}$ configuration and CT excitations $d^{8}\to d^{9}L$ (O-2p→Ni-3d) electron transfer.

NiO is a prototypical strongly correlated TMO and a CT insulator. In stoichiometric NiO, nickel is formally ${\mathrm{Ni}}^{2+}$ with a $3d^{8}$ configuration. In an approximately octahedral NiO_6_ environment, the Ni-3d states can be described by crystal-field splitting into $t_{2g}$ and $e_{g}$ components. However, the insulating gap is not captured by a simple one-electron crystal-field picture and instead reflects substantial electron–electron correlations and strong p−d hybridization [49]. The valence states near the band edge contain a significant O-2p contribution hybridized with Ni-3d, while the unoccupied states have strong Ni-3d character [50] (Figure 10).

### 2.3. Tungsten Oxides

#### 2.3.1. W–O Binary Phase Diagram

The W–O system [51] exhibits one of the most complex phase diagrams among TMOs, characterized by a large number of reported oxide phases spanning a narrow range of oxygen concentrations. This apparent complexity does not arise from the stabilization of numerous independent stoichiometric compounds, but rather from the formation of extended families of structurally related oxygen-deficient and oxygen-rich phases derived from the WO_3_ framework.

In the O-rich region of the W–O phase diagram, the thermodynamic stability is restricted to a limited number of oxide phases. The only fully stoichiometric and widely stable oxides are WO_2_ and WO_3_, which define the lower and upper bounds of oxygen content in this region. Between these two phases, a series of intermediate oxygen-deficient tungsten oxides is stabilized. These phases represent extended homologous series derived from the WO_3_ framework via crystallographic shear mechanisms.

#### 2.3.2. Crystal Structure of Tungsten Oxide WO_3_

**Figure 11:**
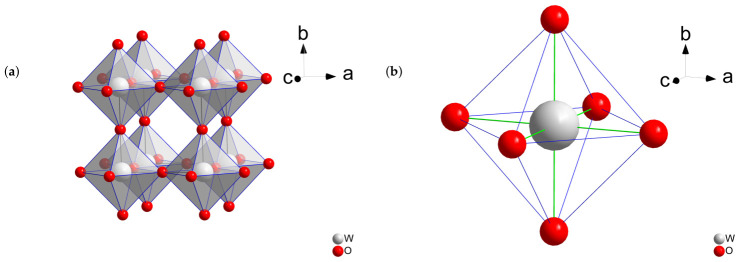
Crystal structure of WO_3_: (**a**) ideal WO_3_ structure in an octahedral environment and (**b**) WO_6_ octahedra.

All polymorphs of WO_3_ can be viewed as distortions of the cubic ${\mathrm{ReO}}_{3}$-type structure $Pm\bar{3}m$ (No. 221) (Table 7, Figure 11a), which consists of a three-dimensional framework of corner-sharing WO_6_ octahedra (Figure 11b).

The ideal cubic WO_3_ structure is metastable and can be stabilized only under non-equilibrium conditions, such as chemical substitution or lattice constraint effects, since the A-site of the perovskite lattice remains vacant. Structural polymorphism in WO_3_ arises primarily from cooperative tilting of WO_6_ octahedra and off-centering of W atoms within the octahedral units [52]. As a result, WO_3_ undergoes a sequence of symmetry-lowering phase transitions with decreasing temperature, exhibiting several stable crystallographic phases over the temperature range from 0 to 1170 K (Table 8).

#### 2.3.3. Electronic Structure of Tungsten Oxide WO_3_

Tungsten trioxide WO_3_ in stoichiometric form is a $d^{0}$ oxide: tungsten is formally in W^6+^ oxidation state with a $5d^{0}$ electronic configuration, so the conduction band is derived primarily from empty W-5d states, whereas the valence band is dominated mainly by O-2p states with significant hybridization [58,59]. In an octahedral environment, the low-lying W-*d* conduction states are often predominantly $t_{2g}$-like, but real structures exhibit sizable distortions that lift degeneracies and modify the band dispersion [60]. WO_3_ exhibits multiple temperature-dependent polymorphs, yet it is generally treated as a semiconductor/insulator in its stoichiometric forms; the key changes are typically in lattice symmetry and quantitative band-edge positions rather than a temperature-driven MIT as in ${\mathrm{VO}}_{2}$ [58,60].

### 2.4. Titanium Oxides

#### 2.4.1. Ti–O Binary Phase Diagram

The Ti–O system can be regarded as an intermediate case among TMOs, combining a relatively high solubility of oxygen in metallic titanium with the formation of numerous non-stoichiometric and shear-structure oxides at increasing oxygen content [61,62].

At intermediate oxygen concentrations, the Ti–O system is dominated by titanium suboxides based on TiO and ${\mathrm{Ti}}_{1-x}$O compositions. The nominal TiO phase crystallizes in a NaCl-type B1 structure and exhibits an exceptionally wide range of non-stoichiometry, which is accommodated by the simultaneous presence of vacancies on both the titanium and oxygen sublattices. As a result, TiO cannot be regarded as a strictly stoichiometric compound, but rather as a defect-stabilized phase with variable composition. Depending on temperature and oxygen chemical potential, TiO-based phases may undergo vacancy ordering, giving rise to lower-symmetry structures such as α-TiO and β-TiO. These suboxides typically exhibit metallic or semimetallic conductivity and represent a transitional regime between metallic solid solutions and oxygen-rich shear-structure oxides. In addition to the nominal TiO composition, the Ti–O system contains several titanium suboxides in the Ti-rich and intermediate oxygen concentration range. Early suboxides such as Ti_3_O, Ti_2_O, and Ti_3_O_2_ are typically interpreted as oxygen-ordered derivatives of metallic titanium rather than distinct stoichiometric oxides.

In the O-rich region of the Ti–O system, phase stability is governed by stoichiometric titanium dioxide TiO_2_ and a series of ordered oxygen-deficient Magnéli phases. The O-rich phases are stabilized by well-defined crystallographic rearrangements of the TiO_6_ octahedral framework.

TiO_2_ represents the fully oxidized and strictly stoichiometric endpoint of the Ti–O system. It is thermodynamically stable over a wide range of temperatures and oxygen partial pressures and crystallizes in several polymorphic modifications, with the rutile structure being the most stable under ambient conditions. All Ti sites in TiO_2_ are occupied by Ti^4+^ ions in octahedral coordination, and large deviations from stoichiometry are energetically unfavorable.

Between TiO and TiO_2_, a series of Magnéli phases with the general formula ${\mathrm{Ti}}_{n}$$O_{2n-1}$ forms a distinct homologous series. These compounds are thermodynamically stable phases rather than defect variants of TiO_2_ and are characterized by the presence of periodic crystallographic shear planes within a rutile-derived structure. The introduction of shear planes accommodates oxygen deficiency without the formation of isolated point defects, resulting in well-defined compositions and long-range structural order. The Magnéli phases exhibit mixed-valence titanium states, Ti^3+^/Ti^4+^, and display electronic properties ranging from semiconducting to metallic, reflecting the progressive reduction of TiO_2_. Structurally and electronically, they constitute a transitional regime between corundum-type Ti_2_O_3_ and rutile TiO_2_, playing a central role in the O-rich phase chemistry of titanium oxides.

#### 2.4.2. Crystal Structure of Titanium Oxide ${\mathrm{TiO}}_{2}$

**Figure 12:**
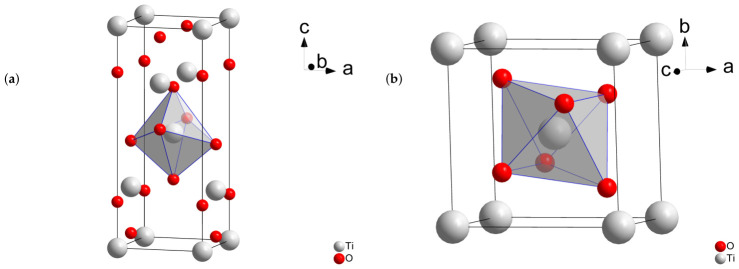
Crystal structure of ${\mathrm{TiO}}_{2}$ (**a**) anatase and (**b**) rutile.

TiO_2_ crystallizes in three main polymorphic phases: anatase, rutile, and brookite. Anatase adopts a tetragonal structure with space group $I4_{1}/amd$ (No. 141) and lattice parameters a=b=3.784Å and c=9.514Å [63] (Table 9, Figure 12a). Rutile crystallizes in the tetragonal space group $P4_{2}/mnm$ (No. 136) with lattice parameters a=b=4.594Å and c=2.959Å [64] (Table 10, Figure 12b), while brookite exhibits an orthorhombic structure belonging to space group Pbca (No. 61) [65,66]. Among these polymorphs, rutile is the thermodynamically stable phase under ambient conditions, whereas anatase and brookite are metastable and can be stabilized depending on synthesis conditions, particle size, and strain.

All polymorphs of TiO_2_ are constructed from TiO_6_ octahedra, in which Ti^4+^ ions are octahedrally coordinated by six $O^{2-}$ ions. The different crystal structures arise from variations in the connectivity, distortion, and tilting of these octahedra. In the rutile structure, TiO_6_ octahedra share edges along the crystallographic c-axis and corners in the perpendicular directions, forming a relatively dense and compact framework. In contrast, anatase exhibits a more open structure with stronger octahedral distortions, which is often associated with enhanced surface reactivity and a higher propensity for defect formation.

#### 2.4.3. Electronic Structure of Titanium Oxide

Titanium dioxide (TiO_2_) is also a prototypical $d^{0}$ oxide: titanium is formally in the Ti^4+^ oxidation state with a $3d^{0}$ electronic configuration. The electronic structure is therefore dominated by O-2p states at the top of the valence band and Ti-3d states at the bottom of the conduction band. In the approximately octahedral TiO_6_ environment, the Ti-3d states are split into lower-energy $t_{2g}$-like and higher-energy $e_{g}$-like groups, although the actual splitting depends on the polymorph, rutile, anatase, or brookite, and on local structural distortions [67,68]. The best-known polymorphs, rutile and anatase, have similar orbital character but differ in quantitative band-structure details, such as band-gap magnitude and band dispersion. For many optical and photochemical effects, a key role is often attributed to oxygen vacancies and related Ti^3+^-associated gap states; modern electronic-structure studies describe their electronic levels using beyond-semilocal DFT approaches, such as hybrid functionals [69].

### 2.5. Comparative Summary of Selected TMOs

Table 11 summarizes the key properties and chromic responses of the selected TMOs, providing the direct comparison between vanadium, nickel, tungsten and titanium oxides.

## 3. Technology of TMO Preparation

### 3.1. Pulsed Laser Deposition

Pulsed laser deposition (PLD) is a physical vapor deposition technique in which a high-power pulsed laser ablates a solid target inside a vacuum chamber, producing a plasma plume that expands toward a substrate where the ablated species condense. The process can be regarded as a sequence of strongly coupled stages: laser–target interaction and ablation, plume formation and expansion, including scattering and gas-phase reactions, and subsequent nucleation and growth on the substrate surface. As a result, the film thickness, microstructure, crystallinity, and phase stability can be tailored by adjusting the laser fluence, repetition rate, substrate temperature, target–substrate distance, and background gas pressure [70,71]. For TMO films, the oxygen background is particularly critical: plume–gas collisions and reactions can promote the formation of oxide species and precursors, enhance oxygen incorporation, and reduce the kinetic energy of arriving species, thereby mitigating surface damage and helping to stabilize the desired phase. In addition, oxygen pressure represents an effective processing parameter for tuning oxygen-related defect populations, such as oxygen vacancies, and consequently the functional properties of the grown oxide [72,73,74].

**Figure 13:**
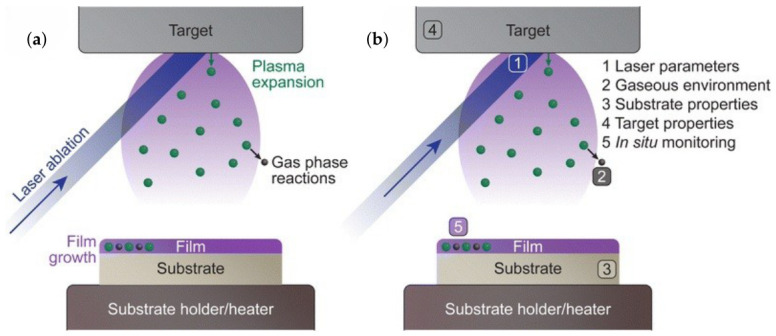
(**a**) An overview of the key processes involved in PLD. (**b**) The main parameters tailored during the PLD process. Reproduced from Shepelin et al. [71] under the CC BY 3.0 license.

A schematic representation of the key PLD processes and main control parameters is shown in Figure 13, while representative deposition conditions for selected TMOs are summarized in Table 12.

A major reason why PLD is widely used for the growth of TMO thin films is its combination of versatility and compositional control. Many complex oxides can be deposited from a single target with the desired composition, and PLD often enables near-stoichiometric transfer because the ablation and transport processes are highly energetic and occur on short time scales. However, deviations between the target and film composition may still occur due to incongruent ablation, preferential scattering in the background gas, and re-sputtering, particularly when the background pressure and kinetic energy of the plume species vary [89]. The energetic, ionized plume can also assist crystalline growth and, in some cases, reduce the substrate temperature required for crystalline or epitaxial film formation. The setup is comparatively clean and convenient for multilayer structures, since different targets can be switched during the same deposition run while the laser source remains outside the chamber [72,73].

At the same time, PLD has well-known limitations that are relevant to practical TMO devices. The plume is typically forward-directed and has a limited cross-sectional area, partly determined by the laser spot size, which complicates thickness uniformity and scaling to large-area substrates. For the same geometrical reason, conformal step coverage and in situ thickness control can also be challenging. Another persistent issue is the formation of droplets and particulates during ablation, which can be incorporated into the film surface and degrade performance or even cause device failure. Thus, PLD is often a method of choice for producing high-quality, compositionally faithful TMO films and multilayer structures, but it requires careful process optimization when thickness uniformity, droplet-free surfaces, and large-area manufacturing are required [71,90].

Overall, the representative deposition conditions summarized in Table 12 highlight the broad tunability of PLD for TMOs, where phase formation and defect populations can be adjusted by oxygen pressure, substrate temperature, and laser parameters. In oxide PLD, KrF excimer lasers (λ=248nm) are used most frequently, and the employed laser fluence is commonly in the 1–$2\;J\;{\mathrm{cm}}^{-2}$ range.

### 3.2. Magnetron Sputtering

Reactive magnetron sputtering is a widely used physical vapor deposition technique for growing TMO thin films with good thickness uniformity and strong industrial scalability. A metallic or suboxide target is bombarded by ions from a plasma, most commonly sustained in argon, ejecting atoms and clusters from the target surface. These sputtered species are transported through the gas phase and condense on a substrate to form a thin film. In the magnetron configuration, a magnetic field near the target confines electrons, enhances ionization, and thereby enables a stable discharge at relatively low pressures and high deposition rates (often with reduced thermal load on the substrate compared to non-magnetron operation) [91,92].

To deposit oxides, O_2_ is introduced together with Ar, and the sputtered metal species react in the plasma and/or at the growing surface to form the oxide. Consequently, phase formation, stoichiometry, defect chemistry, and microstructure are governed by parameters such as total pressure, oxygen partial pressure (or O_2_ flow), Ar:O_2_ ratio, applied power (DC for conductive targets, RF for insulating conditions, and often pulsed-DC in reactive operation), substrate temperature, substrate bias, and the overall plasma composition. For many TMOs, oxygen partial pressure is especially critical because it directly affects oxygen vacancies and therefore electrical and optical properties [93,94].

One of the main advantages of reactive magnetron sputtering for TMO films is its ability to deliver uniformity and reproducibility over large areas. Thus, it is widely used for coatings and device fabrication. It is compatible with a broad range of substrates and can be operated at relatively low substrate temperatures. Multi-target sputtering and co-sputtering further enable composition tuning, doping, and multilayer architectures [93,95].

The method also has limitations. In reactive operation, the target surface can undergo oxidized target poisoning, reducing the deposition rate and introducing process hysteresis, which complicates stable control of oxygen incorporation—especially near the transition between metallic and compound modes [93,94,96].

**Figure 14:**
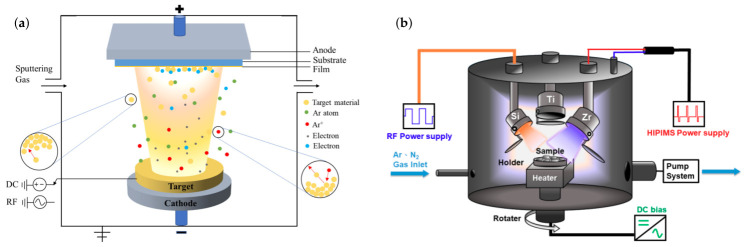
Schematic representation of (**a**) RF–DC magnetron sputtering system. Reproduced from Shahinuzzaman et al. [97] under CC BY 4.0 license. (**b**) High-power impulse magnetron sputtering (HiPIMS) and radio-frequency magnetron sputtering co-sputtering equipment. Reproduced from Chang et al. [98] under the CC BY 4.0 license.

In addition, because the typical kinetic energy of arriving species is lower than in highly energetic plume-based methods, high crystallinity in some TMOs may require elevated substrate temperatures, substrate biasing, or post-deposition annealing. Finally, plasma-driven ion bombardment can induce stress, cause re-sputtering of light elements, or create subsurface damage if pressure and bias conditions are not optimized. Overall, reactive magnetron sputtering is a robust and scalable route to functional TMO thin films, but it requires careful tuning of oxygen and plasma conditions when phase purity, defect engineering, and precise stoichiometry are critical [93,94]. Representative sputtering configurations (RF/DC reactive and HiPIMS) are summarized schematically in Figure 14, while representative growth conditions for selected TMOs are listed in Table 13.

Overall, Table 13 emphasizes the practical versatility of magnetron sputtering for TMO thin films. In most studies, oxide layers are deposited in reactive mode using either DC power, for conductive targets, or RF power, commonly adopted to maintain stable operation when the target surface becomes oxidized. A further advantage is the broad compatibility with substrates of different thermal and structural constraints, including glass and SLG for scalable coatings, together with single-crystal substrates, such as sapphire, when improved crystallinity or texture control is needed. Together with the possibility of multi-target sputtering and co-sputtering, these features make magnetron sputtering an established technique for large-area TMO coatings and device fabrication, where uniformity, reproducibility, and compositional and defect tuning through oxygen control are the primary requirements.

### 3.3. Sol-Gel and Aerosol Spray Deposition

**Figure 15:**
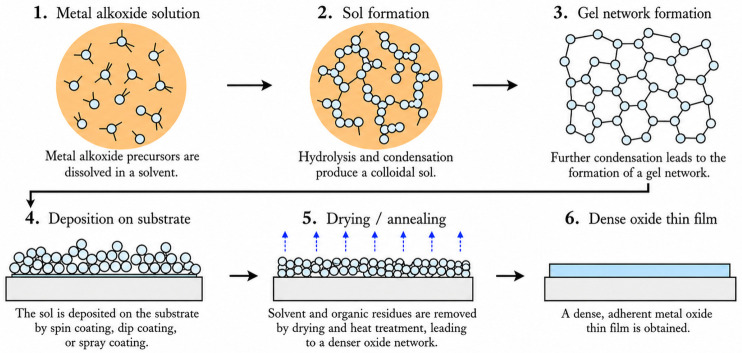
A schematic representation of the sol–gel process.

Sol–gel processing (Figure 15) is a solution-based chemical route for producing inorganic thin films and coatings, in which molecular precursors, commonly metal alkoxides or metal salts, are converted into an extended M–O–M network through hydrolysis and condensation reactions. The resulting colloidal sol evolves toward a gel, and subsequent drying and thermal treatment remove volatile species and organic residues, densify the layer, and, when required, promote crystallization of the final inorganic phase [112].

For thin film formation, the sol is typically deposited onto a substrate by dip-coating or spin-coating, with film thickness and uniformity governed by coupled fluid-dynamic and evaporation phenomena, including withdrawal or spin speed, viscosity, surface tension, and solvent volatility. At the same time, the developing gel network determines shrinkage, porosity, and mechanical integrity during drying. In practice, film properties are strongly influenced by chemical parameters, such as precursor type, solvent system, water-to-precursor ratio, pH, complexing or chelating agents, and aging time, together with processing parameters, including ambient humidity during drying, heating rate, annealing temperature, dwell time, and annealing atmosphere [113].

In aerosol-assisted variants, the precursor sol or solution is atomized into droplets and transported to the substrate, enabling coating of large areas and non-planar geometries and offering a practical pathway toward scalable manufacturing. Depending on temperature and chemistry, conversion to the final inorganic network can occur predominantly after deposition, as in aerosol-gel or aerosol-assisted sol–gel processing, or partially during flight, impact, and subsequent heating, as in spray-based routes [114,115,116]. Representative TMO thin film examples obtained using sol–gel routes and their processing conditions are summarized in Table 14.

Overall, Table 14 highlights the practical versatility of sol–gel processing for TMO thin films. Across the reported studies, spin coating and dip coating are the most common deposition modes, while a wide variety of substrates, including Si, quartz, glass, and transparent conductive glass such as ITO- or FTO-coated glass, can be employed depending on the targeted device architecture. The cited examples also illustrate that sol–gel chemistry provides multiple precursor options, including alkoxides, acetylacetonates, chlorides, and acetates, as well as straightforward composition tuning at the solution-processing stage. Importantly, the final oxide phase, crystallinity, and defect-related properties are largely determined by the drying and annealing steps, including atmosphere control, making thermal processing a central processing parameter for sol–gel prepared TMOs in functional coatings.

### 3.4. Other Deposition Methods

Beyond PLD, magnetron sputtering, and sol–gel, several other thin film technologies are frequently employed to access either (i) highly conformal coatings and precise thickness control, (ii) scalable conformal growth on complex geometries, or (iii) epitaxial films for fundamental structure–property studies. In the context of the present TMOs (VO_2_, WO_3_, TiO_2_, NiO), particularly relevant routes include atomic layer deposition (ALD), chemical vapor deposition (CVD, including aerosol-assisted CVD), electrochemical growth (electrodeposition/anodization), and molecular beam epitaxy (MBE).

Atomic layer deposition (ALD) is especially attractive when thickness control and conformality on textured or 3D substrates are required. VO_2_ films have been demonstrated by ALD using inorganic precursors, such as ${\mathrm{VCl}}_{4}$, with direct crystallization under suitable conditions [129]. NiO electrochromic coatings have also been prepared by ALD on transparent conductive substrates, illustrating the viability of ALD for anodic electrochromic oxides [130]. For WO_3_, ALD processes, such as $W{\left(\mathrm{CO}\right)}_{6}+O_{3}$, have been reported as a route to controlled ultrathin tungsten oxide films [131]. TiO_2_ is one of the best-established ALD oxides; comprehensive reviews summarize its wide precursor chemistry and broad application space [132].

CVD and aerosol-assisted CVD (AACVD) methods are widely used for scalable oxide coatings, often providing good step coverage and industrial compatibility. Thermochromic VO_2_ films have been fabricated by low-pressure CVD (LPCVD), demonstrating controllable growth of crystalline VO_2_ from volatile vanadium precursors [133]. AACVD has been used to prepare doped VO_2_ films as well as WO_3_ coatings on glass from solution-deliverable precursors [134,135]. For NiO, metal–organic methods have been reported for electrochromic NiO coatings, highlighting that gas-phase chemistry can also be leveraged for this anodic electrochromic oxide [136].

Electrochemical methods, including electrodeposition and anodization, are particularly common for electrochromic TMOs because they naturally integrate with conductive substrates and can yield porous or high-surface-area morphologies. NiO electrochromic films have been produced by electrodeposition onto FTO-coated glass [136]. WO_3_ is likewise frequently prepared by electrodeposition for electrochromic applications, with multiple studies and reviews treating electrodeposition as a key low-cost method to obtain functional WO_3_ coatings [137]. For TiO_2_, anodization of Ti or Ti-coated substrates is a standard method to obtain self-organized TiO_2_ nanotube arrays, enabling strongly nanostructured films after subsequent annealing [138,139].

Molecular beam epitaxy (MBE), including oxide or oxygen-assisted variants, is often used when epitaxial, well-defined model films are needed to probe intrinsic mechanisms and substrate/strain effects. VO_2_ ultrathin films have been grown by reactive MBE on rutile TiO_2_ substrates [140]. Plasma-assisted MBE has also been applied to epitaxial NiO films on semiconducting substrates [141]. For WO_3_, single-crystalline and epitaxial WO_3_ thin films grown by MBE have been reported and used to study orientation-dependent functional behavior [142,143]. Epitaxial rutile TiO_2_ films on sapphire grown by MBE have been reported, illustrating MBE control over phase and microstructure [144].

Overall, other routes complement PLD, sputtering, and sol–gel processing by offering either superior conformality and thickness control (ALD), scalable conformal growth with strong industrial relevance (CVD/AACVD), device-friendly electrochemical fabrication of porous electrochromic layers (electrodeposition/anodization), or high-purity epitaxial model films for mechanistic studies (MBE).

### 3.5. Comparative Analysis of TMO Thin Film Preparation Technologies

The preparation route is a key factor that determines not only the phase composition, crystallinity, morphology, and stoichiometry of chromic TMO thin films, but also their technological relevance for practical devices. While laboratory-scale methods such as PLD and MBE are highly useful for obtaining well-controlled model systems and clarifying structure–property relationships, scalable techniques such as magnetron sputtering, sol–gel processing, spray deposition, ALD, CVD, and electrochemical deposition are more directly connected with device fabrication. Therefore, the choice of deposition method should be evaluated not only in terms of film quality, but also with respect to scalability, processing temperature, substrate compatibility, reproducibility, cost, and integration into multilayer or device architectures. A comparative overview of the main advantages, limitations, and device-integration aspects of the reviewed preparation technologies is summarized in Table 15.

## 4. Devices and Applications

### 4.1. Thermochromic VO_2_-Based Smart Windows

SWs are a critical component of the building envelope because they simultaneously manage (i) transmission heat losses driven by temperature differences, (ii) solar heat gains driven by short-wave irradiance, and (iii) daylight access, which affects both lighting energy demand and visual comfort.

In the energy balance of a building, the contribution of a window is commonly described by two main terms: a non-solar term, determined by the overall heat transfer coefficient (*U*-value), and a solar term, determined by the solar heat gain coefficient (SHGC, or *g*-value). At the same time, optical parameters such as visible transmittance ($T_{\mathrm{lum}}$) and solar transmittance ($T_{\mathrm{sol}}$) govern the quality of daylighting and solar-energy admission. Therefore, window performance is inherently multi-objective: a low *U*-value and an optimized SHGC are central design goals, but they must be balanced against daylight access, outside view, condensation resistance, and the long-term durability of the insulating glass unit (IGU) [145].

In more detailed analyses, the angular dependence of solar transmission and the spectral transmittance and reflectance over the solar spectrum are also taken into account. In typical double glazing with an air-filled cavity, a substantial part of the heat transfer across the gap may occur through long-wave radiative exchange between the panes, especially because uncoated glass has high thermal emittance. In addition, perimeter components such as spacers and edge seals may act as thermal bridges and strongly affect service life by limiting moisture ingress and fill-gas leakage. These edge-region effects are therefore not secondary: they directly influence the effective window-level $U_{w}$ and determine whether the initial thermal performance is maintained over time [146].

A conventional window is typically characterized by standardized performance parameters, including U∼0.35, SHGC∼0.8, $T_{\mathrm{vis}}∼0.9$, and $T_{\mathrm{sol}}∼0.8$.

A passive way to improve glazing performance is to combine low-emissivity (low-e) coatings with optimized cavity fills. Low-e coatings suppress long-wave radiative heat transfer by lowering the surface emissivity of the glazing, thereby reducing $U_{g}$ without necessarily sacrificing high visible transmittance $T_{\mathrm{vis}}$ when spectrally selective designs are used. Reviews of commercially available low-e materials and products show that such coatings are already a widely implemented strategy for improving both the thermal and optical performance of windows [147]. In parallel, replacing air in the cavity with inert gases, most commonly Ar and, in higher-performance configurations, Kr, reduces conductive and convective heat transfer across the gap. However, the magnitude and long-term persistence of this benefit depend strongly on the quality of the edge seal and spacer design, since these components govern both gas retention and resistance to moisture ingress over service life [146].

An active way to further improve glazing performance beyond low-e coatings and gas fills includes triple glazing, warm-edge spacers, vacuum insulating glazing, and smart glazing technologies that dynamically tune solar and/or visible transmittance to match operational needs. Reviews show that electrochromic and related SWs can reduce cooling demand by modulating transmittance under external control. They can also lower lighting demand by maintaining a more favorable daylighting regime. In contrast, thermochromic systems, often based on VO_2_, provide passive, temperature-driven solar control. The physics and optimization of thermochromic performance, particularly the trade-off between solar modulation and luminous transmittance, have been extensively reviewed and experimentally characterized [148].

**Figure 16:**
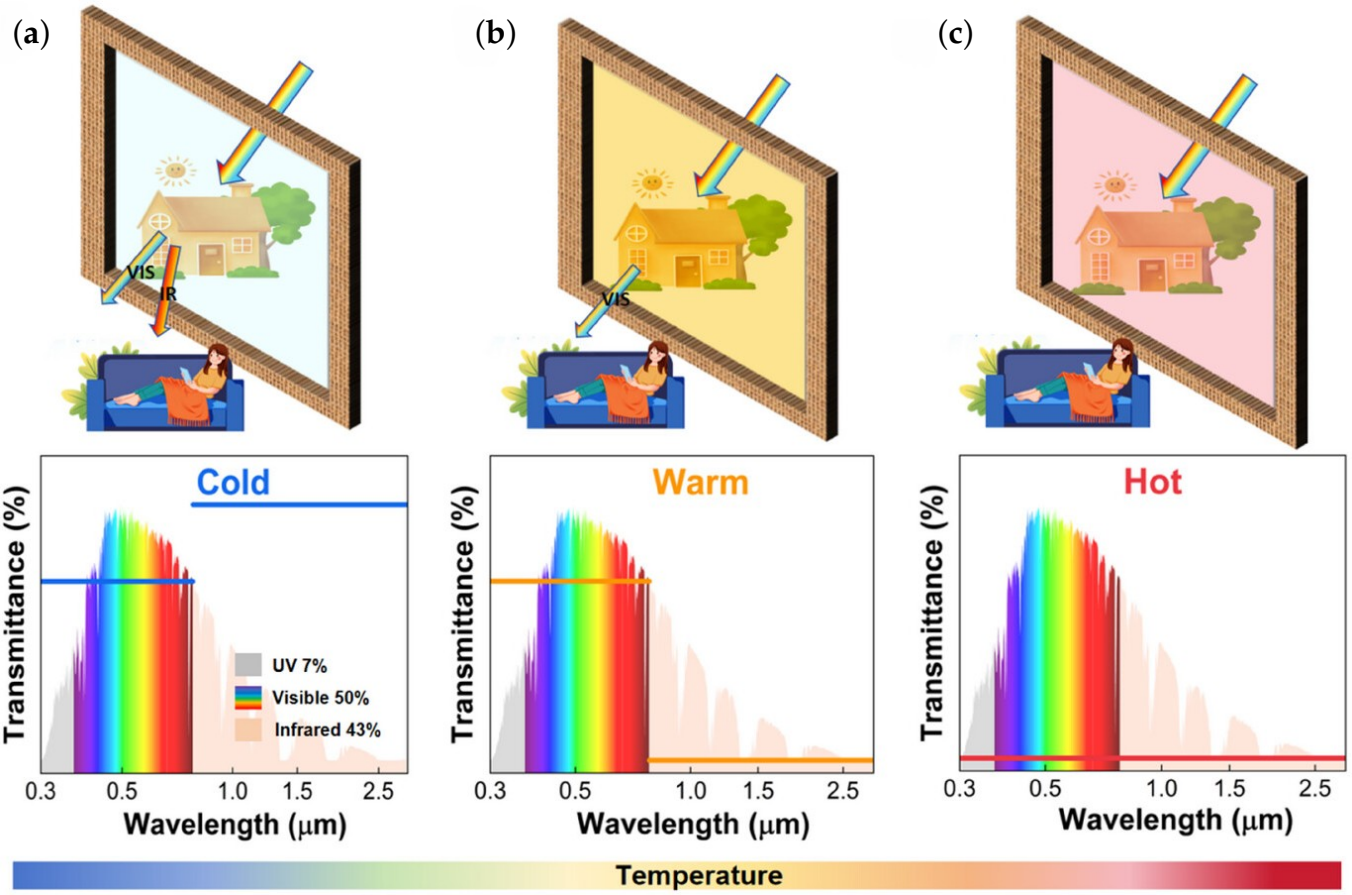
Ideal three-state thermochromic SW. The blue, yellow, and red lines represent the spectra for an ideal energy-saving SW in the (**a**) cold state, (**b**) warm state, and (**c**) hot state. Reproduced from Liu et al. [149] under the CC BY 4.0 license.

In summer, an ideal SW (Figure 16) should maintain a bright indoor environment while limiting solar heat gains. It should transmit most of the visible light, about 60–80% in the 380–780 nm range, to preserve daylighting and a clear outside view, while blocking or reflecting a significant portion of the near-infrared radiation, about 780–2500 nm, which is mainly responsible for solar heating. Thus, an SW should remain highly transparent in the visible range, suppress NIR transmission, and, ideally, facilitate net radiative heat rejection from the indoor side to the outdoor environment.

In winter, an ideal SW should maximize useful solar heat gains while minimizing heat losses. It should still maintain high visible transmittance, about 60–80% in the 380–780 nm range, to ensure daylight access, but it should also exhibit high solar transmittance over a broad part of the solar spectrum to support passive heating. In the long-wave infrared, the desirable behavior is generally the opposite of the summer case: the window should reduce radiative heat loss by reflecting thermal radiation emitted by indoor surfaces back into the room. Thus, the winter-optimal state combines good visible transparency and enhanced solar admission with improved retention of indoor thermal radiation.

The application of vanadium oxide in SW is limited by its intrinsic properties, namely relatively low luminous transmittance ($T_{\mathrm{lum}}∼40\%$), relatively high absorption, and a limited solar modulation efficiency ($\Delta T_{\mathrm{sol}}\leq 10\%$) for a single layer. Moreover, the critical temperature ($T_{c}∼68$ °C) is too high for most climatic regions. Therefore, lowering the critical temperature to $T_{c}\leq 25\;{}^{\circ}$C and improving $T_{\mathrm{lum}}$ are the main requirements for practical VO_2_-based SWs. Another challenge is the long-term cycling stability of VO_2_, since in the presence of oxygen it may undergo further oxidation to V_2_O_5_, which is the most stable oxidation state among vanadium oxides. These challenges can be addressed by doping, morphology engineering, and multilayer design.

Elemental doping is one of the conventional approaches used to tailor the MIT temperature and optical properties (Table 16). The choice of dopant is usually guided by two main considerations: increasing the carrier density to promote the electronic phase transition, or inducing lattice distortion to facilitate the structural phase transition [150].

**Figure 17:**
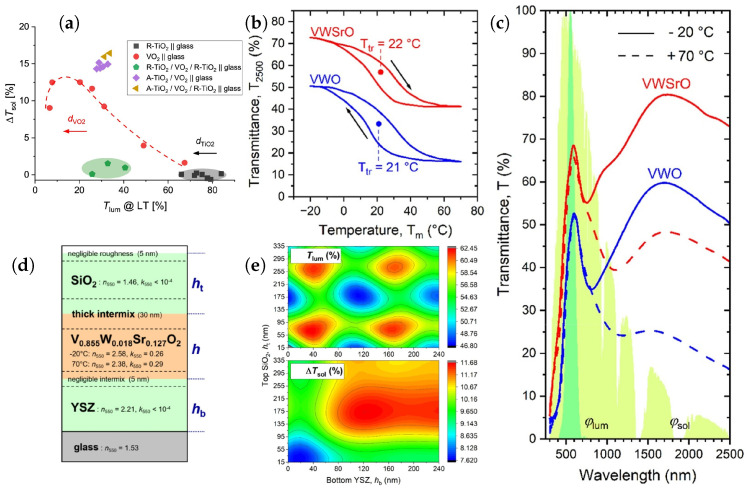
Optical optimization of ${\mathrm{VO}}_{2}$-based SW coatings. (**a**) $\Delta T_{\mathrm{sol}}$ as a function of $T_{\mathrm{lum}}$ at low temperature for different ${\mathrm{TiO}}_{2}/{\mathrm{VO}}_{2}$ multilayer configurations. Reproduced from Becker et al. [164] under the CC BY 4.0 license. (**b**) Temperature-dependent transmittance at 2500nm, (**c**) spectral transmittance at −20°C and +70°C, (**d**) multilayer coating design, and (**e**) calculated $T_{\mathrm{lum}}$ and $\Delta T_{\mathrm{sol}}$ maps as a function of YSZ and ${\mathrm{SiO}}_{2}$ layer thicknesses. Reproduced from Kaufman et al. [165] under the CC BY 4.0 license.

Besides lowering $T_{c}$, another major challenge for practical VO_2_-based SWs is to achieve sufficiently high luminous transmittance ($T_{\mathrm{lum}}$) together with adequate solar modulation ability ($\Delta T_{\mathrm{sol}}$). One possible solution is related to the design and architecture of the glazing stack. A VO_2_-based SW typically consists of a multilayer thin film structure, as shown in Figure 17. To reduce optical reflection losses and increase luminous transmittance, additional antireflection (AR) layers may be incorporated into the structure (Figure 17d). Candidate AR materials are listed in Table 17 and can be selected depending on the target optical and functional requirements of the device. The optimum thickness and the appropriate refractive index of these layers can be evaluated by means of the transfer matrix method (TMM), which is widely used for the optical design and optimization of multilayer coatings. Thus, multilayer engineering and the integration of AR coatings provide an effective route for improving the optical performance of VO_2_-based methods.

Thus, a practical route toward high-performance thermochromic glazing is obtained by combining two complementary optimization strategies: chemical tuning of the active VO_2_ by doping and optical tuning of the complete coating stack by AR, buffer, and protective layers. Doping mainly controls the intrinsic thermochromic response of VO_2_, especially the $T_{c}$, visible absorption, and NIR modulation, whereas AR and buffer layers control reflection losses, optical interference, crystallization, interfacial strain, and long-term stability. Therefore, the most successful VO_2_-based SW coatings are multilayer structures in which composition, thickness, morphology, refractive-index, and interface quality are optimized simultaneously [164,165,166].

Becker et al. [164] investigated the role of TiO_2_ AR and buffer layer in undoped VO_2_ film in order to separate the effect of optical multilayer engineering from doping of the VO_2_ layer. The choice of TiO_2_ is motivated by the following considerations. Rutile TiO_2_ is structurally compatible with the high-temperature rutile phase of VO_2_ and can therefore promote the formation of the desired thermochromic phase, whereas TiO_2_ top layers can act as AR coatings for reducing reflection losses and increasing $T_{\mathrm{lum}}$. In this concept, the bottom rutile TiO_2_ layer acts mainly as a buffer layer, while the top anatase TiO_2_ layer acts as an AR layer. The coating was prepared by ion-beam sputter deposition (IBSD) on quartz glass, using a customized ceramic TiO_2_ target for the deposition of both rutile and anatase TiO_2_ layers and a metallic V target with a purity of 99.9% for the VO_2_ layer. This technique was selected because it allows the sequential deposition of oxide thin films under controlled energetic ion bombardment, which is important for stabilizing the desired TiO_2_ and VO_2_ phases and for controlling the morphology and interface quality of the multilayer stack. In the first step, R-TiO_2_, VO_2_, and VO_2_/R-TiO_2_ films were deposited on quartz glass at 550°C, providing the reference structures required to evaluate the influence of the rutile TiO_2_ buffer layer on the growth and thermochromic response of VO_2_. The optical constants, namely the refractive index *n* and extinction coefficient *k*, were determined by spectroscopic ellipsometry at 25°C and 90°C, corresponding to the low- and high-temperature states of VO_2_. These experimentally determined optical constants were then used in Macleod simulations to optimize the thicknesses of the R-TiO_2_ buffer layer and the A-TiO_2_ AR layer.

For a fixed VO_2_ thickness of 50 nm, Becker et al. calculated the thermochromic parameters as a function of the rutile-TiO_2_ buffer thickness and the anatase-TiO_2_ AR thickness in the range 0–300 nm. The optimized ideal architecture was obtained for a rutile-TiO_2_ buffer thickness of approximately 152 nm and an anatase-TiO_2_ AR thickness of approximately 178 nm, giving predicted enhancements of about 8% in $\Delta T_{\mathrm{sol}}$ and about 25% in $T_{\mathrm{lum}}$ compared with a bare VO_2_ layer on quartz glass. Experimentally, the realized trilayer coating improved both $T_{\mathrm{lum}}$ and $\Delta T_{\mathrm{sol}}$ by approximately 5–8%. However, the authors showed that using rutile TiO_2_ as the top AR layer is problematic, because the oxygen-rich and high-temperature conditions required for rutile growth may oxidize the underlying VO_2_ toward higher vanadium oxidation states, including V_2_O_5_. For this reason, anatase TiO_2_ was selected as the top AR layer, since it can be deposited under milder conditions while still providing AR and capping functions.

A key conclusion of Becker et al. is that ideal optical simulations overestimate the real performance if interface roughness is neglected. In the ideal case, the optimized multilayer stack gave a simulated $T_{\mathrm{lum}}$ of about 45%, whereas the inclusion of effective-medium interface layers with thicknesses of about 4–5 nm at the rutile-TiO_2_/VO_2_ interface and about 8–10 nm at the VO_2_/anatase-TiO_2_ interface reduced the calculated $T_{\mathrm{lum}}$ to approximately 35–37%, in better agreement with experiment. Thus, Becker et al. demonstrated that TiO_2_ buffer and AR layers can improve the optical response of VO_2_-based coatings, but also that AR engineering alone is insufficient to reach the practical SW requirements of $T_{\mathrm{lum}}>60\%$ and a transition temperature close to room temperature [164] (Figure 17a).

A more complete approach was reported by Kaufman et al., who combined controlled W/Sr codoping of VO_2_ with a second-order AR multilayer design [165]. The active layer composition was $V_{0.855}$$W_{0.018}$${\mathrm{Sr}}_{0.127}$O_2_ (Figure 17d). W was chosen because it is one of the most effective dopants for reducing the $T_{c}$ of VO_2_, mainly by destabilizing the low-temperature semiconducting phase and increasing the electron concentration. Sr was chosen because it improves the visible optical properties by widening the visible-range optical gap and lowering the extinction coefficient in the visible spectral range. In particular, the incorporation of 12.7at.% Sr into the metal sublattice increased the visible-range gap $E_{g1}$ from 1.51 to 1.75 eV, decreased $n_{550}$ from 2.84–3.13 to 2.38–2.58, and decreased $k_{550}$ from 0.45–0.47 to 0.29–0.26. These changes explain why Sr strongly increases $T_{\mathrm{lum}}$, while W keeps the transition temperature near room temperature.

The optimized coating architecture reported by Kaufman et al. [165] was YSZ (167 nm)/$V_{0.855}$$W_{0.018}$${\mathrm{Sr}}_{0.127}$O_2_ (71 nm)/SiO_2_ (280 nm) on 1 mm SLG. The coating was prepared by reactive high-power impulse magnetron sputtering (HiPIMS) at a substrate temperature of 320°C and without substrate bias voltage. The top SiO_2_ layer was selected because Sr lowers the refractive index of the active layer, making the optimum top-AR refractive index about 1.54–1.61, which is much closer to SiO_2_ with $n_{550}\approx 1.46$ than to high-index oxides such as YSZ with $n_{550}\approx 2.21$.

Kaufman et al. deliberately used second-order interference in the AR layers rather than only maximizing the first-order visible-transmittance maximum. For a thermochromic layer thickness of 71 nm, optical modeling gave optimum AR thicknesses close to ∼170 nm for YSZ and ∼269 nm for SiO_2_, which is consistent with the experimentally realized 167 nm/71 nm/280 nm structure. This design is important because the second-order maximum in the visible region can be combined with a favorable first-order maximum in the infrared region, thereby improving $\Delta T_{\mathrm{sol}}$ without sacrificing too much $T_{\mathrm{lum}}$. The same modeling also showed the trade-off associated with the thermochromic layer thickness: increasing the $V_{0.855}$$W_{0.018}$${\mathrm{Sr}}_{0.127}$O_2_ thickness from 40 to 100 nm decreases the predicted $T_{\mathrm{lum}}$ from 72.6% to 49.6%, but increases the predicted $\Delta T_{\mathrm{sol}}$ from 7.41% to 13.61% (Figure 17e). Thus, the selected thickness of 71 nm represents a compromise between high visible transmittance and strong solar modulation.

The optimized W/Sr-codoped coating achieved $T_{c}=22\;{}^{\circ}$C, $T_{\mathrm{lum}}=63.7\%$ in the low-temperature state, $T_{\mathrm{lum}}=60.7\%$ in the high-temperature state, $T_{\mathrm{sol}}=58.8\%$ in the low-temperature state, $T_{\mathrm{sol}}=47.6\%$ in the high-temperature state, and $\Delta T_{\mathrm{sol}}=11.2\%$. For comparison, the W-only reference coating YSZ (178 nm)/$V_{0.984}$$W_{0.016}$O_2_ (73 nm)/SiO_2_ (280 nm) showed only $T_{\mathrm{lum}}=47.0\%$ and 45.3% in the low- and high-temperature states, respectively, with $\Delta T_{\mathrm{sol}}=8.4\%$. Therefore, Sr doping increased the low-temperature $T_{\mathrm{lum}}$ by 16.7% and the high-temperature $T_{\mathrm{lum}}$ by 15.4%, while also increasing $\Delta T_{\mathrm{sol}}$ by 2.8% (Figure 17c). This result shows that W/Sr codoping combined with YSZ/SiO_2_ AR design can simultaneously satisfy the practical thresholds of $T_{\mathrm{lum}}>60\%$, $\Delta T_{\mathrm{sol}}>10\%$, and $T_{c}$ close to room temperature.

A further important contribution in this direction was reported by Vlček et al. [166], who demonstrated that the thermochromic performance of VO_2_-based coatings can be significantly improved by replacing the compact active layer with W-doped VO_2_ nanoparticles embedded in a SiO_2_ matrix. W was again selected to reduce the $T_{c}$, while the nanoparticle morphology was selected to reduce the effective visible-range refractive index and extinction coefficient. The SiO_2_ matrix and overlayer were chosen because SiO_2_ has a low refractive index, n≈1.46 at λ = 550 nm, negligible absorption with $k<10^{-3}$ at λ = 550 nm, and can simultaneously act as a Na-diffusion barrier, low-index matrix, AR medium, and protective layer. In addition, the metallic VO_2_(R) nanoparticles can enhance NIR absorption through localized surface plasmon resonance, which increases $\Delta T_{\mathrm{sol}}$ without excessively suppressing visible transmittance.

The coating reported by Vlček et al. was prepared on 1 mm SLG at a maximum substrate temperature of 350°C. The structure consisted of a bottom ∼100 nm SiO_2_ layer blocking Na diffusion from the glass substrate, four W-doped VO_2_ nanoparticle layers separated by three ∼50 nm SiO_2_ layers, and a top ∼160 nm SiO_2_ AR/protective overlayer. Each nanoparticle layer was obtained by depositing a ∼10 nm V–W film, annealing it up to 350°C for 1 h in pure oxygen at $p_{O_{2}}=15$ Pa, cooling below 50°C, and depositing the next SiO_2_ layer. The V–W films were deposited from a V–W target containing 4.0wt.% W, corresponding to 1.14at.% W, and the W-induced $T_{c}$ gradient was at least −24°C/at.% W.

**Figure 18:**
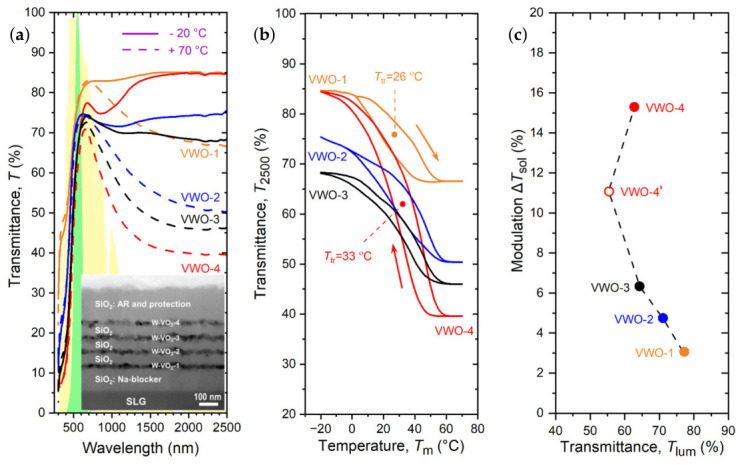
Optical and thermochromic performance of multilayer VWO coatings for SWs applications. (**a**) Spectral transmittance of thermochromic coatings with 1, 2, 3, and 4 WVO layers measured at −20°C and +70°C, with the visible spectral region highlighted; the inset shows the cross-sectional structure of the multilayer coating. (**b**) Temperature-dependent transmittance at 2500nm for VWO-2, VWO-3, and VWO-4 coatings, showing the thermochromic transition behavior. (**c**) Solar modulation $\Delta T_{\mathrm{sol}}$ as a function of luminous transmittance $T_{\mathrm{lum}}$ for the investigated VWO coatings. Reproduced from Vlček et al. [166] under the CC BY 4.0 license.

TEM and HRTEM showed W-doped VO_2_ nanoparticles embedded in the SiO_2_ matrix, with representative vertical nanoparticle sizes of about 42 nm in the upper W–VO_2_ layer and about 22 nm in the lower W–VO_2_ layer (Figure 18a inset). SEM analysis showed that most nanoparticles had diameters of 41–55 nm and interparticle spacings of 7–21 nm, while some elongated particles reached lengths of 150–400 nm or even about 1 μm.

The gradual increase in the number of W-doped VO_2_ layers produced the expected trade-off between visible transmittance and solar modulation. For one, two, three, and four nanoparticle layers with approximately 50 nm SiO_2_ overlayers, $\Delta T_{\mathrm{sol}}$ increased from 3.1% to 4.7%, 6.3%, and 11.1%, respectively, while the low-temperature $T_{\mathrm{lum}}$ decreased from 76.3% to 71.9%, 64.9%, and 56.8%. Replacing the ∼50 nm top SiO_2_ overlayer in the four-layer coating with an optimized ∼160 nm top SiO_2_ AR/protective layer strongly improved the performance. The final VWO-4 coating reached $T_{\mathrm{lum}}=65.4\%$ at low temperature, $T_{\mathrm{lum}}=60.1\%$ at high temperature, $T_{\mathrm{sol}}=66.1\%$ at low temperature, $T_{\mathrm{sol}}=50.8\%$ at high temperature, $\Delta T_{\mathrm{lum}}=5.3\%$, $\Delta T_{\mathrm{sol}}=15.3\%$, and $T_{c}=33\;{}^{\circ}$C [166]. This is the highest $\Delta T_{\mathrm{sol}}$ among the three selected approaches while still maintaining $T_{\mathrm{lum}}>60\%$ in both thermochromic states (Figure 18).

Overall, these studies illustrate a clear progression in VO_2_-based SW design. Becker et al. [164] demonstrated that buffer and AR layers can improve the optical performance of undoped VO_2_, but the resulting $T_{\mathrm{lum}}$ remains limited by the intrinsic absorption of the active layer and by interface roughness. Kaufman et al. solved this limitation more effectively by combining W/Sr codoping with second-order YSZ/SiO_2_ AR design, reaching $T_{c}=22\;{}^{\circ}$C, $T_{\mathrm{lum}}>60\%$, and $\Delta T_{\mathrm{sol}}>10\%$ in a compact multilayer coating prepared at 320°C [165]. Vlček et al. achieved the highest solar modulation by introducing W-doped VO_2_ nanoparticles into a SiO_2_ matrix, where the combination of reduced effective optical constants, localized near-infrared absorption, and optimized SiO_2_ AR/protection produced $\Delta T_{\mathrm{sol}}=15.3\%$ while preserving $T_{\mathrm{lum}}>60\%$ [166]. Thus, the most promising direction is the simultaneous optimization of dopant chemistry, optical interference, layer morphology, and interface stability rather than the use of a single modification strategy.

In comparison with VO_2_ and other inorganic thermochromic material systems, PNIPAm-based hydrogels generally show superior optical performance. PNIPAm-AEMA hydrogel microparticles have shown $T_{\mathrm{lum}}=87.2\%$, $\Delta T_{\mathrm{sol}}=81.3\%$, and a lower critical solution temperature (LCST) of around 32°C [173]. HBPEC/PNIPAm hydrogels reached $T_{\mathrm{lum}}=87.5\%$, $\Delta T_{\mathrm{sol}}=71.2\%$, and a tunable transition range of 24.1–33.2°C [174]. PNIPAm/HEMC hydrogels were also reported with $T_{\mathrm{lum}}\approx 85\%$ and $\Delta T_{\mathrm{sol}}\approx 75\%$ [149]. These values are substantially higher than those of most VO_2_ coatings, for which $\Delta T_{\mathrm{sol}}$ typically remains below 20%. However, hydrogel systems face different device-integration challenges, including encapsulation, water retention, drying/freezing resistance, mechanical stability during swelling/deswelling, and long-term outdoor durability.

Emerging thermochromic systems occupy an intermediate position between VO_2_ and hydrogel-based systems. Metal-halide perovskite SWs based on CH_3_NH_3_PbI_3_ can exhibit visible transmittance above 85% in the cold state and about 34.3% in the hot state, with $\Delta T_{\mathrm{sol}}=25.5\%$ and a transition temperature of approximately 54.4°C [175]. Hydrated ionic polymers, such as ${\left[{\left(C_{2}H_{5}\right)}_{2}{\mathrm{NH}}_{2}\right]}_{2}{\mathrm{NiCl}}_{4}$@PVP-type systems, combine $T_{\mathrm{lum}}=87.7\%$, $\Delta T_{\mathrm{sol}}=30.5\%$, and a tunable transition temperature of 25–42°C [176]. Commercial IR-selective thermochromic coatings, such as SunSmart coatings, show more moderate but application-oriented values, with $T_{\mathrm{lum}}\approx 70$–75% and $\Delta T_{\mathrm{sol}}\approx 20$–23% [177]. Cholesteric liquid-crystal thermochromic films are promising for selective NIR modulation and composition-tunable optical response, although their performance metrics are less standardized than those of VO_2_ or PNIPAm-based systems [178].

These comparisons indicate that the most effective SW designs will likely rely on combining the strengths of different material classes rather than selecting a single universal material system. VO_2_ offers inorganic stability, compatibility with thin film processing, and strong NIR switching, but it still requires simultaneous improvement of $T_{c}$, $T_{\mathrm{lum}}$, $\Delta T_{\mathrm{sol}}$, color neutrality, interface quality, and scalable deposition. Hydrogels provide the highest optical performance, with $T_{\mathrm{lum}}>85\%$ and $\Delta T_{\mathrm{sol}}>70\%$, but their long-term durability and encapsulation remain unresolved. Perovskites and hydrated ionic polymers offer high contrast and tunable transition temperatures, but they require further validation with respect to toxicity, humidity resistance, UV stability, hysteresis, and cycling lifetime. Commercial IR-selective coatings are closer to real glazing implementation, but their optical modulation is lower than that of laboratory hydrogel systems.

### 4.2. Electrochromic WO_3_- and NiO-Based Smart Windows

Among electrochromic materials for SW applications, TMOs remain the most extensively investigated class because they combine reversible optical modulation, chemical robustness, thermal stability, and compatibility with conventional thin film fabrication methods. In TMO-based electrochromic SWs, the optical response is governed by electrically driven redox reactions coupled with the insertion/extraction of ions, such as $H^{+}$, ${\mathrm{Li}}^{+}$, ${\mathrm{Na}}^{+}$. Therefore, the electrochromic performance is determined not only by the intrinsic optical contrast of the active oxide, but also by ion-diffusion kinetics, CT reversibility, electrolyte compatibility, and long-term cycling stability.

WO_3_ serves as the benchmark cathodic electrochromic oxide. Upon application of a suitable voltage, electrons are injected into the WO_3_ layer while charge-compensating ions from the electrolyte are inserted into its structure, forming a colored tungsten-bronze-like state, generally described as $M_{x}$WO_3_, where M represents the inserted ion. When the voltage polarity is reversed, ions and electrons are extracted and the material returns to its bleached transparent state. In contrast, NiO is the prototypical anodic oxide counterpart, where coloration is associated with oxidation of Ni^2+^ toward higher-valence nickel states, such as Ni^3+^ and Ni^4+^. Consequently, the complementary integration of cathodic WO_3_ and anodic NiO remains one of the standard configurations for high-contrast electrochromic SWs.

**Figure 19:**
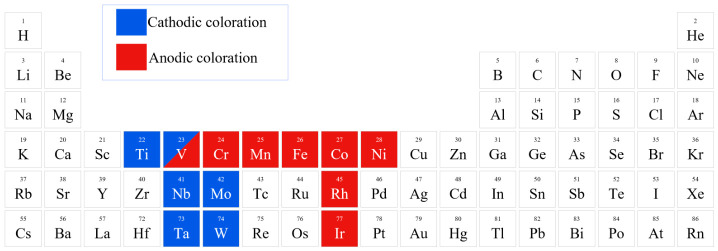
Cathodic and anodic type coloration of TMOs.

Besides the benchmark WO_3_/NiO pair, other TMOs can also contribute to electrochromic functionality. V_2_O_5_ is attractive because its layered framework enables ion intercalation, multicolor switching, and infrared modulation, although structural stabilization is often required to improve cycling durability. TiO_2_, especially in anatase or nanostructured form, is frequently employed either as a weak active electrochromic component or, more importantly, as an ion-storage, passivation, or interfacial layer in all-inorganic and multilayer electrochromic architectures. TMOs are commonly classified into cathodically coloring and anodically coloring materials, depending on the polarity and redox process responsible for coloration [179] (Figure 19).

**Figure 20:**
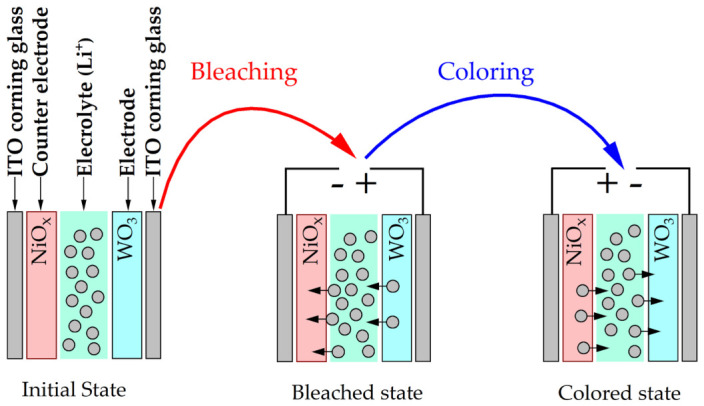
Design of a typical electrochromic SW.

A typical electrochromic SW consists of two transparent substrates coated with transparent conducting layers, between which an electrochromic layer, an ion-conducting electrolyte, and a complementary counter-electrode or ion-storage layer are arranged in a sandwich-type architecture. Under an applied voltage, ions migrate through the electrolyte toward one electrode, while electrons are supplied through the external circuit. In the colored state, ion/electron insertion into the cathodic layer, most commonly WO_3_, increases optical absorption in the VIS and/or NIR region. At the same time, the anodic counter electrode, typically NiO or a Ni-containing oxide, balances the charge and can provide complementary coloration. Reversing the voltage extracts the inserted ions and electrons, restoring the bleached state. Therefore, the optical modulation of an electrochromic SW is a device-level response controlled by the active oxide layers, the transparent conductors, the electrolyte, and the stability of their interfaces (Figure 20).

The electrolyte controls ionic conductivity, switching speed, charge compensation, optical memory, leakage resistance, and interfacial degradation. Liquid and gel electrolytes generally provide high ionic conductivity and faster switching, but may suffer from leakage, evaporation, gas formation, and sealing problems. In contrast, solid polymer, inorganic, and hybrid electrolytes are more attractive for all-solid-state laminated windows because they improve mechanical integrity and device stability, although their ionic conductivity can be lower. Typical electrolyte types used in TMO-based electrochromic windows are summarized in Table 18.

TMOs practical relevance cannot be assessed only from the maximum optical contrast. Recent WO_3_ nanosheet/ITO nanocomposites reached optical modulation values up to ΔT≈88% and a coloration efficiency (CE) of $154.16\;{\mathrm{cm}}^{2}\;C^{-1}$ [184]. Rare-earth-modified WO_3_ also shows strong performance: Tb-doped amorphous WO_3_ exhibited an optical contrast of 78.34%, a CE of $138.82\;{\mathrm{cm}}^{2}\;C^{-1}$, and coloration/bleaching times of 16.0/11.4s [185]. These values are high enough to justify the continued dominance of WO_3_ in electrochromic SW research, especially when compared with many other oxide systems. Nevertheless, WO_3_ suffers from the classical limitations of cathodic oxide electrochromics: repeated H^+^ or Li^+^ insertion/extraction can produce ion trapping, defect accumulation, local structural disorder, incomplete bleaching, and gradual loss of optical reversibility. Therefore, a WO_3_ film showing ΔT>70% in the first cycles is not necessarily suitable for practical glazing unless the same modulation is retained over thousands of cycles.

Recent improvements in WO_3_-based devices clearly show that interface engineering is more effective than simple single-layer optimization. For example, WO_3_/TiO_2_ electrochromic devices achieved ΔT=73.9% at 600nm, CE up to $69\;{\mathrm{cm}}^{2}\;C^{-1}$, coloration times of about 20–21s, and stable operation over more than 1000 cycles [186]. More advanced TiO_2_/WO_3_/TiO_2_ double-heterojunction structures achieved a much higher CE of $479.3\;{\mathrm{cm}}^{2}\;C^{-1}$ and retained 94.72% of their transmission modulation after 7000 cycles under recovery conditions [187]. This comparison is important because the CE of the TiO_2_/WO_3_/TiO_2_ heterostructure is more than three times higher than that of the WO_3_ nanosheet/ITO nanocomposite and more than six times higher than the WO_3_/TiO_2_ device. Although TiO_2_ alone is usually a weak visible electrochromic material, in multilayer devices it acts as an ion-storage, passivation, or interfacial layer that suppresses irreversible ion accumulation and improves CT reversibility.

NiO is the most important anodic oxide counterpart to WO_3_ in complementary electrochromic devices. Recent W^6+^-doped NiO films showed an optical modulation of 61.56% at 550nm, a CE of $45.41\;{\mathrm{cm}}^{2}\;C^{-1}$, fast switching times of 4.42/1.40s for coloration/bleaching, and stable operation over 2000 cycles [188]. These figures indicate that NiO can switch faster than many WO_3_ systems, but its CE remains lower than that of optimized WO_3_, Prussian Blue, PANI, or PEDOT:PSS. Thus, NiO is not generally selected because it provides the highest CE, but because it is one of the most suitable anodic charge-balancing layers for WO_3_-based full devices. Its main limitations are its relatively low electrical conductivity, moderate CT kinetics, and degradation during prolonged cycling, which explains why doping, defect engineering, and nanostructuring are required for practical SW integration.

Alternative Ni-based compounds can provide stronger optical modulation than conventional NiO. Electrodeposited ${\mathrm{NiHPO}}_{4}\cdot$3H_2_O films demonstrated an optical modulation of 90.8% at 500nm and a CE of $75.4\;{\mathrm{cm}}^{2}\;C^{-1}$, while also enabling a large-area electrochromic device with an active area of $100\;{\mathrm{cm}}^{2}$ [189]. The ΔT value of 90.8% is higher than that reported for the WO_3_ nanosheet/ITO system, Tb-doped WO_3_, W-doped NiO, V_2_O_5_, PANI, PEDOT:PSS, and viologen/PEDOT:PSS/Zn devices considered here. From a manufacturing perspective, electrodeposition is also attractive because it is lower-cost and more scalable than pulsed laser deposition or high-vacuum multilayer deposition. However, the long-term outdoor durability of nickel phosphate under humidity, UV exposure, thermal cycling, and realistic electrolyte conditions still requires further validation.

TiO_2_ should be considered separately from WO_3_ and NiO because its role in SWs is often interfacial rather than purely electrochromic. TiO_2_-based heterostructures, such as 2D TiO_2_/Ti_3_$C_{2}T_{x}$ MXene systems, have been reported to provide high optical modulation, fast response, and good mechanical stability in flexible devices [190]. In Au nanoparticle-loaded TiO_2_ nanofibrous layers, the optical modulation was approximately ΔT≈40%, the CE was about $20\;{\mathrm{cm}}^{2}\;C^{-1}$, the switching time was around 6s, and the devices operated for more than 1000 cycles [191]. These values are lower than those of optimized WO_3_ or conducting-polymer systems, but TiO_2_ remains highly valuable because it can improve cycling stability, protect active layers, assist ion storage, and enable flexible or nanostructured device architectures.

V_2_O_5_ is attractive because it offers multicolor electrochromism and very fast switching. Annealed V_2_O_5_ films showed a bleached transmittance of $T_{b}=71.89\%$, a colored transmittance of $T_{\mathrm{col}}=29.57\%$, an optical modulation of ΔT=42.32%, a CE of $34.93\;{\mathrm{cm}}^{2}\;C^{-1}$, and very short coloration/bleaching times of 0.4/3.0s [192]. The switching speed of V_2_O_5_ is therefore better than that of many WO_3_-based devices and comparable to fast polymer systems. However, its optical contrast can degrade significantly after only about 100 cycles, which is far below the 2000 cycles reported for W-doped NiO, the 7000 cycles reported for TiO_2_/WO_3_/TiO_2_, and the 10,000 cycles reported for PANI-based devices. This makes V_2_O_5_ promising for rapid or multicolor electrochromic functions, but still problematic for long-lifetime architectural glazing.

When TMOs are compared with non-oxide electrochromic materials, the differences become even clearer. Nanogranular Prussian Blue films reached optical modulation values of about 80%, CEs as high as $417.79\;{\mathrm{cm}}^{2}\;C^{-1}$, and 91.4% optical contrast retention [193]. Thus, the CE of Prussian Blue is approximately 2.7 times higher than that of the WO_3_ nanosheet/ITO system, about 9.2 times higher than that of W-doped NiO, and about 12 times higher than that of V_2_O_5_. This demonstrates the strong charge-utilization advantage of Prussian Blue, although its dominant blue coloration and electrolyte sensitivity can limit its use in color-neutral architectural glazing.

Conducting polymers also show highly competitive electrochromic performance. Polyaniline-based dual-band electrochromic SWs demonstrated ΔT=65% at 633nm and ΔT=59% at 1600nm, with CEs of $367.1\;{\mathrm{cm}}^{2}\;C^{-1}$ in the visible region and $299.6\;{\mathrm{cm}}^{2}\;C^{-1}$ in the NIR region, together with stability exceeding 10,000 cycles [194]. This is particularly important because dual-band modulation allows independent control of visible light and solar heat, a functionality that most single-component oxide systems cannot easily provide. PEDOT:PSS-based devices achieved an optical contrast of 47.9% at 635nm, a CE of $352\;{\mathrm{cm}}^{2}\;C^{-1}$, and very fast switching times of 1.6/0.6s [195]. Although the optical contrast of PEDOT:PSS is lower than that of WO_3_, Prussian Blue, or PANI, its switching speed and solution processability make it attractive for printed, flexible, and low-temperature electrochromic devices.

Viologen-based hybrid systems further illustrate the advantages of organic electrochromics. Viologen/PEDOT:PSS/Zn devices reached an optical difference of 64.23%, a CE of $389.44\;{\mathrm{cm}}^{2}\;C^{-1}$, switching times of 1.6/0.8s, and 90.58% retention of ΔT [196]. The CE of this hybrid system is higher than that of WO_3_, NiO, nickel phosphate, TiO_2_, and V_2_O_5_, and its switching is faster than most oxide systems. However, aqueous organic–metal hybrid devices may suffer from swelling, electrolyte leakage, dissolution, interfacial instability, and degradation of organic redox species during prolonged outdoor exposure. Therefore, their excellent short-term electrochemical performance must be balanced against environmental durability requirements.

A direct performance comparison therefore shows that each material class has a distinct advantage. WO_3_ provides high optical modulation, with values up to ΔT≈88%, and remains the most reliable cathodic oxide. NiO provides fast anodic switching, with 4.42/1.40s switching times and 2000-cycle stability, but lower CE. TiO_2_ improves interfaces and cycling stability rather than acting as the strongest active electrochromic layer. V_2_O_5_ provides very fast switching, down to 0.4s for coloration, but poor cycling stability. Nickel phosphate provides very high optical modulation of 90.8% and scalable electrodeposition. Prussian Blue, PANI, PEDOT:PSS, and viologen systems provide much higher CE values, typically in the range of 352–$417.79\;{\mathrm{cm}}^{2}\;C^{-1}$, but their environmental stability is generally more problematic than that of inorganic oxides.

From the viewpoint of deposition and manufacturing, TMOs are compatible with magnetron sputtering, PLD, ALD, sol–gel processing, and electrodeposition. Vacuum-based methods such as sputtering and ALD offer dense, uniform, and reproducible coatings, which are essential for large-area optical uniformity, but they increase equipment cost and may limit low-cost manufacturing. PLD is useful for high-quality research films but is less attractive for industrial-scale glazing. Sol–gel processing and electrodeposition are more scalable and cost-effective, as illustrated by the $100\;{\mathrm{cm}}^{2}$ nickel phosphate device, but they require strict control of film porosity, adhesion, crystallinity, and post-treatment. Processing temperature is also critical: high-temperature annealing can improve oxide crystallinity and cycling stability, whereas low-temperature processing is required for flexible substrates, polymers, and laminated window structures.

At the device level, electrolyte compatibility is as important as the active electrochromic material. H^+^, Li^+^, Na^+^, Zn^2+^, and other mobile ions can provide electrochromic switching, but they also determine diffusion kinetics, reversibility, charge compensation, interfacial stability, and degradation pathways. Dense WO_3_ films may suffer from slow ion diffusion and ion trapping, whereas organic and hydrogel-like devices may suffer from swelling, leakage, or dissolution. A device with high CE is therefore not automatically superior if the electrolyte causes gas evolution, parasitic redox reactions, delamination, or rapid loss of optical memory. For practical SWs, the target is not simply the highest ΔT or CE, but the best combination of optical contrast, response speed, cycling lifetime, color neutrality, haze control, sealing, and compatibility with large-area laminated glazing.

These comparison shows that no single electrochromic material currently maximizes all relevant parameters. WO_3_ remains the benchmark cathodic oxide because it provides high optical modulation and relatively strong durability. NiO remains the preferred anodic oxide because it enables complementary WO_3_-based devices. TiO_2_ is most valuable as an interfacial and stabilizing component. V_2_O_5_ is attractive for rapid and multicolor switching but is limited by cycling degradation.

For commercial applications, ITO, FTO, AZO, and PEDOT or PEDOT-based transparent conducting coatings are widely used.

At present, a relatively large number of both major and smaller companies are involved in the production of electrochromic SW. Table 19 summarizes selected commercial products from four companies chosen for this review, as they represent different technological, commercial, and manufacturing approaches within the SW sector. A common feature of these products is the use of a WO_3_/NiO cathode–anode configuration.

However, the exact layer chemistry used in commercial electrochromic SWs is rarely disclosed in current product literature and is generally treated as proprietary. Publicly available information is therefore often fragmentary and must be reconstructed from patents, older technical brochures, and review papers. Taken together, these sources suggest that many commercially relevant inorganic electrochromic SW platforms rely on a tungsten-oxide-based cathodic layer combined with a nickel-oxide-based, nickel-containing, or mixed tungsten–nickel anodic/counter electrode, frequently in lithiated or otherwise compositionally modified form rather than as strictly stoichiometric WO_3_ and NiO. Accordingly, the systems listed in Table 19 are best described as devices that, with a high degree of confidence, appear to belong to the broader WO_3_/NiO materials family.

### 4.3. Optical Hydrogen Detection Using TMO-Based Gasochromic Sensors

Hydrogen leakage detection is a critical safety requirement because $H_{2}$ [197]: (i) is flammable over a wide concentration range in air, typically ∼4–75vol.%; (ii) exhibits an extremely low minimum ignition energy, 0.017mJ; (iii) is colorless, odorless, and tasteless, making leaks difficult to notice without instrumentation; (iv) can cause hydrogen embrittlement of structural materials, thereby increasing the probability of failures and leakage.

Gasochromic hydrogen sensors [197] provide an optical route to leak detection by converting the gas–solid interaction into a reversible change of the materials’ optical response, such as transmittance, reflectance, absorbance, or perceived color. In TMO materials, the gasochromic response is commonly attributed to near-surface redox reactions, intercalation–deintercalation, which result in increased absorption in the VIS–NIR region. Gasochromic hydrogen sensors have several key features: (i) they can operate at room temperature and may not require external electrical biasing at the sensing element, reducing ignition risks; (ii) hydrogen exposure can induce a distinct optical contrast, including visible color changes in some systems, enabling direct visual indication and straightforward optical readout; (iii) the device concept can be implemented using simple thin film stacks and low-cost optical interrogation, e.g., photodiodes or cameras, which is attractive for scalable safety indicators.

Among TMOs, tungsten trioxide WO_3_ is widely applicable as one of the most practically established materials for optical hydrogen detection [197]. This preference is largely driven by its pronounced and reversible optical modulation upon hydrogen exposure, typically a pale-yellow to deep-blue coloration [198,199]. In most device implementations, WO_3_ is combined with a thin Pd or Pt catalytic overlayer to dissociate H_2_ and promote spillover/insertion, enabling large changes in transmittance with good repeatability [200]. These features make Pd/Pt–WO_3_ coatings a common choice for passive, optically readable hydrogen-leak indicators [199,201].

Vanadium oxides represent another important gasochromic TMO class for hydrogen optical sensing [197]. In particular, V_2_O_5_ thin films, often combined with a noble-metal catalyst such as Pt, exhibit a pronounced and reversible optical contrast upon H_2_ exposure under ambient conditions, e.g., from yellow to gray/black, consistent with hydrogen-driven redox processes [202,203,204]. In parallel, VO_2_ is frequently exploited in a distinct route, where the gas environment, hydrogenation, shifts the MIT and produces a large change in resistance [205,206]. The MIT in VO_2_ is accompanied by a strong modulation of optical constants, especially in the NIR/MIR region, so a gas-induced MIT shift can, in principle, be read out optically as well, either as a gasochromic contrast or as an MIT-assisted transmittance/reflectance change [207].

In contrast to WO_3_ and several vanadium oxides, NiO [208] and TiO_2_ [209] are most commonly deployed as chemiresistive metal oxide (MOX) sensors, where the primary signal is a gas-induced change in conductivity rather than a pronounced, reversible optical contrast. Nevertheless, both oxides can still be integrated into optically readable sensor architectures at the device level. For NiO, its well-known anodic electrochromism under ion intercalation–deintercalation can be leveraged as a visual transducer in electrochromic sensor concepts, where a gas-driven electrochemical/potentiometric process is converted into a measurable optical modulation of the NiO layer [8,210]. For TiO_2_, a practical route toward optical/low-temperature operation is photoactivated sensing, where UV illumination modifies surface charge chemistry and enables room-temperature or reduced-temperature gas detection, with optical control and straightforward optical interrogation schemes [209,211]. Finally, TiO_2_ can develop Ti^3+^ or oxygen-vacancy centers under strongly reducing, hydrogenating treatments, producing blue/black optical states. This suggests a possible gas-induced optical pathway, although this typically requires elevated temperatures and careful engineering to ensure reversibility and selectivity [212,213].

## 5. Future Perspectives

The development of chromic TMO thin films is moving from the optimization of individual films toward the design of complete device architectures. The central issue is the simultaneous control of optical, ionic, electronic, mechanical, and environmental parameters within the same structure.

For thermochromic VO_2_, the relevant performance window combines $T_{c}$ close to 20–35°C, $T_{\mathrm{lum}}>60\%$, $\Delta T_{\mathrm{sol}}>10$–15%, narrow hysteresis, and stable optical response during repeated heating and cooling. For electrochromic devices, the technologically meaningful range combines ΔT>60–80%, $CE>100\;{\mathrm{cm}}^{2}\;C^{-1}$ where attainable, coloration/bleaching below 10–20s at device scale, and optical-modulation retention above 90% after 5000–10,000 cycles. These combined targets are more restrictive than isolated record values and therefore represent a more realistic criterion for SW integration.

VO_2_ demonstrates the balance between material improvement and optical trade-off. W/Sr-codoped VO_2_ multilayers can reduce $T_{c}$ to 22°C while maintaining $T_{\mathrm{lum}}=63.7\%$ in the low-temperature state and 60.7% in the high-temperature state, with $T_{\mathrm{sol}}$ decreasing from 58.8% to 47.6% and $\Delta T_{\mathrm{sol}}=11.2\%$ [165]. W-doped VO_2_/SiO_2_ nanoparticle coatings reach $T_{\mathrm{lum}}=65.4\%$ and 60.1% in the low- and high-temperature states, $\Delta T_{\mathrm{sol}}=15.3\%$, and $T_{c}=33\;{}^{\circ}C$ [166].

AI and machine-learning approaches provide a route for treating chromic oxides as multi-parameter systems. In NiO, recent machine-learning studies mainly concern optical-property prediction in RF-magnetron-sputtered or doped films [214,215]. Machine learning has been used to connect WO_3_ sputtering parameters with electrochromic reversibility and blue-color persistence [216]. In an ammonium metatungstate/FeCl_2_/D_2_O a multilayer perceptron model trained on 25 devices enabled a device with ΔT=74%, coloration and bleaching times of 6.5 and 13.5s, and stability above 1000 cycles [217]. Another machine-learning-guided all-liquid electrochromic device reached ΔT=62.6% with response times of 5.7 and 7.1s [218]. Long short-term memory (LSTM) modelling has also been applied to cycle-life prediction, giving ΔT=43.95%, coloration/bleaching times of 7 and 8s, and ΔT=44.92% after 1000 cycles [219]. Overall, AI is useful for device and multilayer structure optimization, and also for property prediction.

Interface engineering represents a decisive factor in both thermochromic and electrochromic oxide devices. In VO_2_ multilayers, a few nanometers of interfacial roughness can change the optical response substantially. For TiO_2_/VO_2_/TiO_2_ stacks, an ideal model predicted $T_{\mathrm{lum}}$ of about 45%, whereas the introduction of effective-medium interface layers of only 4–5nm and 8–10nm reduced the calculated $T_{\mathrm{lum}}$ to approximately 35–37% [164]. Thus, nominal layer thickness is not sufficient for describing real optical stacks. Interface roughness, interdiffusion width, oxidation-state gradients, and the optical constants of the actual multilayer determine the final coating performance [6,164]. In electrochromic devices, WO_3_/electrolyte, NiO/electrolyte, TiO_2_/WO_3_, and transparent-conductor/oxide interfaces control CT resistance, ion trapping, incomplete bleaching, and long-term drift [186,220,221]. The high CE reported for TiO_2_/WO_3_/TiO_2_ heterostructures shows that TiO_2_ can operate as an active interfacial component that improves CT reversibility and suppresses irreversible ion accumulation [186,187].

Nanostructuring is effective only when optical gain, ion transport, and durability remain balanced. In W-doped VO_2_/SiO_2_ nanoparticle coatings, increasing the number of nanoparticle layers from one to four increased $\Delta T_{\mathrm{sol}}$ from 3.1% to 11.1%, but decreased low-temperature $T_{\mathrm{lum}}$ from 76.3% to 56.8% [166]. After optimization of the SiO_2_ AR/protective layer, the coating reached $T_{\mathrm{lum}}=65.4\%$, $\Delta T_{\mathrm{sol}}=15.3\%$, and $T_{c}=33\;{}^{\circ}C$ [166]. In electrochromic WO_3_, NiO, and V_2_O_5_, nanosheets, porous films, nanofibers, and nanocomposites reduce ion-diffusion length and increase electrochemically active surface area [182,184,222,223,224]. At the same time, excessive porosity and uncontrolled open morphology can increase electrolyte penetration, local structural distortion, irreversible ion trapping, incomplete ion extraction, and cycling degradation [11,192,222]. V_2_O_5_ demonstrates this trade-off clearly: coloration and bleaching times of 0.4 and 3.0s are very fast, but degradation after about 100 cycles remains incompatible with long-life smart-window operation [192].

Flexible and large-area devices introduce constraints that are not visible in small-area cells. Commercial electrochromic glazing already reaches dimensions of several square meters (Table 19) In contrast, many laboratory TMO devices are tested on areas of only a few ${\mathrm{cm}}^{2}$. Scaling from ${\mathrm{cm}}^{2}$ to $m^{2}$ changes the dominant limitations: sheet resistance, lateral voltage drop, electrolyte distribution, edge sealing, defect density, and non-uniform coloration become central performance factors [180,225]. Flexible devices add another set of variables, including bending radius, number of bending cycles, ΔT retention after bending, adhesion loss, electrolyte leakage, and change in sheet resistance [226,227]. Optical modulation measured before bending is therefore insufficient for evaluating flexible electrochromic devices, because mechanical stability, electrochemical reversibility, electrode conductivity, and encapsulation integrity must also be retained after repeated deformation [182,190].

Overall, AI-assisted optimization, flexible processing, interface engineering, and nanostructuring are connected strategies for achieving reproducible device performance under realistic optical, electrochemical, mechanical, and environmental conditions.

## 6. Conclusions

Chromic TMO thin films are promising materials for adaptive optical technologies, including SWs, optical switching, gas sensing, and multifunctional coatings. The systems discussed in this review, namely VO_2_ and related vanadium oxides, WO_3_, NiO, and TiO_2_, show that chromic behavior is controlled by the interplay between crystal structure, electronic configuration, oxidation state, oxygen stoichiometry, defects, ion transport, and thin film microstructure. Thus, their optical response must be understood not only from chemical composition, but also from phase purity, morphology, interfaces, and preparation conditions.

VO_2_ remains the reference thermochromic oxide because its MIT transition enables passive temperature-driven optical modulation, especially in the NIR region. WO_3_ and NiO are key electrochromic oxides, with performance governed by ion insertion/extraction, charge compensation, crystallinity, porosity, electrolyte compatibility, and cycling stability. V_2_O_5_ extends chromic functionality toward multicolor electrochromism and gas sensing, whereas TiO_2_ is important as a defect-active oxide and as an auxiliary layer in electrochromic, photocatalytic, antireflective, and optoelectronic architectures.

The preparation route is a decisive factor for chromic performance. Deposition and post-treatment conditions control thickness, stoichiometry, crystallinity, morphology, defects, and interfaces, which directly affect optical contrast, switching kinetics, stability, and reproducibility. Therefore, efficient chromic coatings require the simultaneous optimization of material composition, thin film processing, and device architecture.

Despite significant progress, several challenges remain. VO_2_-based systems still require better control of transition temperature, hysteresis, visible transparency, solar modulation, and long-term stability. WO_3_-, NiO-, V_2_O_5_-, and TiO_2_-based systems are often limited by ion-transport constraints, charge trapping, slow switching, structural fatigue, and cycling degradation. These limitations are especially important for SW applications, where high luminous transmittance, strong solar modulation, color neutrality, durability, and large-area manufacturability must be achieved simultaneously.

Future research should focus on the following directions:

– improving VO_2_ thermochromic coatings through reducing the transition temperature while preserving high luminous transmittance, strong solar modulation, narrow hysteresis, and environmental stability through doping, strain engineering, multilayer design, protective layers, and nanostructuring;
– enhancing WO_3_/NiO-based electrochromic oxide devices, namely faster switching, lower operating voltage, higher optical contrast, better color neutrality, and longer cycling lifetime through improved ion transport, electrolyte compatibility, charge balance, and interface stability;
– controlling defects and microstructure such as oxygen vacancies, mixed-valence states, grain boundaries, and porosity to tune optical modulation, ion diffusion, switching kinetics, and degradation;
– expanding gasochromic WO_3_ and V_2_O_5_ devices for hydrogen sensing and optical gas detection through improving sensitivity, selectivity, reversibility, humidity tolerance, and stable low-temperature operation.

Overall, future TMO films should be compatible with scalable deposition methods, large-area and flexible substrates, and multilayer architectures, while combining chromism with antireflection, photocatalysis, sensing, thermal regulation, or energy-related functions.

## Figures and Tables

**Table 1:** VO_2_ M1 atomic parameters [21].

Atom	Wyckoff	Site Symmetry	x/a	y/b	z/c
V1	4e	1	0.23947	0.97894	0.02646
O2	4e	1	0.10616	0.21185	0.20859
O3	4e	1	0.40051	0.70258	0.29884

**Table 2:** VO_2_ rutile atomic parameters [22].

Atom	Wyckoff	Site Symmetry	x/a	y/b	z/c
V1	2a	m.mm	0	0	0
O2	4f	m.2m	0.305	0.305	0

**Table 3:** V_2_O_3_ corundum structure atomic parameters [25].

Atom	Wyckoff	x/a	y/b	z/c
V1	12c	0	0	0.34629
O2	18e	0.3118	0	0.25

**Table 4:** V_2_O_3_ monoclinic structure atomic parameters [26].

Atom	Wyckoff	x/a	y/b	z/c
V1	8f	0.3445	0.0025	0.3001
O1	8f	0.4043	0.8493	0.6459
O2	18e	0.25	0.3166	0.5

**Table 5:** V_2_O_5_ atomic parameters [27].

Atom	Wyckoff	x/a	y/b	z/c
V	4f	0.10118	0.25	0.8917
O1	4f	0.1043	0.25	0.531
O2	4f	−0.0689	0.25	0.003
O3	2a	0.25	0.25	0.001

**Table 6:** NiO atomic parameters [48].

Atom	Wyckoff	x/a	y/b	z/c
Ni1	4a	0	0	0
O2	4b	1/2	1/2	1/2

**Table 7:** Ideal cubic WO_3_ structure atomic parameters.

Atom	Wyckoff	x/a	y/b	z/c
W1	1a	0	0	0
O2	3b	1/2	0	0

**Table 8:** ${\mathrm{WO}}_{3}$ stable phases.

Phase	Structure	Lattice Parameters	Stability Temperature Range
α–WO_3_ [53]	Tetragonal P4/nmm	a=5.250Å	1010–1170K
	(No. 129)	c=3.910Å	
β–WO_3_ [54]	Orthorhombic Pnma	a=7.340Å	600–1010K
	(No. 62)	b=7.570Å	
		c=7.750Å	
γ–WO_3_ [8]	Monoclinic $P2_{1}/n$	$a=7.300\;Å\;\beta =90.88^{\circ }$	290–600K
	(No. 14)	b=7.540Å	
		c=7.690Å	
δ–WO_3_ [55]	Triclinic $P\bar{1}$	$a=7.300\;Å\;\alpha =88.81^{\circ }$	230–290K
	(No. 2)	$b=7.510\;Å\;\beta =90.985^{\circ }$	
		$c=7.681\;Å\;\gamma =90.985^{\circ }$	
ϵ–WO_3_ [56]	Monoclinic Pc	$a=5.277\;Å\;\beta =91.76^{\circ }$	<230 K
	(No. 7)	b=5.155Å	
		c=7.663Å	
H-WO_3_ [57]	Hexagonal P6/mmm	a=7.298Å	metastable
	(No. 191)	c=7.798Å	

**Table 9:** Anatase ${\mathrm{TiO}}_{2}$ atomic parameters [63].

Atom	Wyckoff	x/a	y/b	z/c
Ti1	4a	0	0	0
O2	8eb	0	0	0.20806

**Table 10:** Rutile ${\mathrm{TiO}}_{2}$ atomic parameters [64].

Atom	Wyckoff	x/a	y/b	z/c
Ti1	2a	0	0	0
O2	4f	0.306	0.306	0

**Table 11:** Comparative summary of key properties of selected TMOs.

Properties	Vanadium Oxides	Nickel Oxides	Tungsten Oxides	Titanium Oxides
Stoichiometry/ Oxidation state	– VO_2_; V^4+^; $3d^{1}$	– NiO; Ni^2+^; $3d^{8}$	– WO_3_; W^6+^; $5d^{0}$	– TiO_2_; Ti^4+^; $3d^{0}$
	– V_2_O_3_; V^3+^; $3d^{2}$	– ${\mathrm{Ni}}_{1-x}$O;	– ${\mathrm{WO}}_{3-x}$;	– ${\mathrm{TiO}}_{2-x}$;
	– V_2_O_5_; V^5+^; $3d^{0}$	Ni vacancies;	W^5+^/W^6+^ states	oxygen vacancies;
	– $V_{n}$$O_{2n-1}$/	Ni^3+^ states		Ti^3+^ states
	$V_{n}$$O_{2n+1}$; mixed-valence phases			– ${\mathrm{Ti}}_{n}$$O_{2n-11}$; Ti^3+^/Ti^4+^ Magnéli phases
Crystal Structure/ Main Phases	VO_2_: monoclinic M1 ($P2_{1}/c$, No. 14);	NiO: rock-salt ($Fm\bar{3}m$, No. 225)	α-WO_3_: tetragonal (P4/nmm, No. 129);	TiO_2_: anatase ($I4_{1}/amd$, No. 141);
	VO_2_: monoclinic M2 (C2/m, No. 12);		β-WO_3_: orthorhombic (Pnma, No. 62);	TiO_2_: rutile ($P4_{2}/mnm$, No. 136)
	VO_2_: rutile R ($P4_{2}/mnm$, No. 136);		γ-WO_3_: monoclinic ($P2_{1}/n$, No. 14);	TiO_2_: brookite (Pbca, No. 61)
	V_2_O_3_: corundum ($R\bar{3}c$, No. 167);		δ-WO_3_: triclinic ($P\bar{1}$, No. 2);	
	V_2_O_3_: monoclinic (I2/a, No. 15);		ϵ-WO_3_: monoclinic (Pc, No. 7);	
	V_2_O_5_: orthorhombic (Pmmn, No. 59)		H-WO_3_: hexagonal (P6/mmm, No. 191)	
Electronic Structure	VO_2_: correlated oxide; V-3d $t_{2g}$ states split into $a_{1g}$ and $e_{g}^{\pi }$; temperature-driven MIT	CT/correlated insulator; O-2p states dominate the valence band;	wide-band-gap $d^{0}$ oxide; O-2p valence band and W-5d conduction band;	wide-band-gap $d^{0}$ oxide; O-2p valence band and Ti-3d conduction band;
	V_2_O_3_: strongly correlated oxide; $t_{2g}$ states split into $a_{1g}$ and $e_{g}^{\pi }$ by trigonal distortion; Mott–Hubbard-type MIT	Ni-3d states contribute to unoccupied states and multiplet/CT excitations	electron/ion insertion creates W^5+^/W^6+^ states	oxygen vacancies and Ti^3+^ states introduce defect levels
	V_2_O_5_: insulating CT oxide; O-2p valence band and V-3d conduction band;			
Main Chromic Mechanism	VO_2_: Peierls-Mott MIT & V_2_O_3_: Mott MIT → thermochromism;	deintercalation/hole-polaron formation → anodic electrochromism > Alexandru V.:	intercalation/electron-polaron formation → cathodic electrochromism and gasochromism	photoinduced charge trapping/small-polaron formation → photochromism
	V_2_O_5_: intercalation/deintercalation → electrochromism/gasochromism			

**Table 12:** PLD deposition conditions for selected transition-metal oxide films.

TMO	Substrate	PLD Parameters	Laser Type and Parameters
${\mathrm{VO}}_{2}$	Pt(111)/${\mathrm{TiO}}_{2}$ [75]	Substrate temperature: 600°C Distance to substrate: 50mm Working pressure $p_{O_{2}}:\;∼1\;\mathrm{Pa}$ Target: $V_{2}O_{5}$	KrF excimer Frequency: 5Hz Fluence: $1.0\;J\cdot {\mathrm{cm}}^{-2}$ Deposition time: –
	Si/${\mathrm{SiO}}_{2}$ [76]	Substrate temperature: 600°C Distance to substrate: 31mm Working pressure $p_{O_{2}}:\;∼6.67\;\mathrm{Pa}$ Target: V-metal	KrF excimer Frequency: 10Hz Fluence: $2.6\;J\cdot {\mathrm{cm}}^{-2}$ Deposition time: 15min
	Soda lime glass (SLG) [77]	Substrate temperature: 450–600°C Distance to substrate: 65mm Working pressure $p_{O_{2}}:\;∼2\;\mathrm{Pa}$ Target: V-metal	KrF excimer Frequency: 10Hz Fluence: $1.7\;J\cdot {\mathrm{cm}}^{-2}$ Deposition time: 45min
	c-plane sapphire [78]	Substrate temperature: 600°C Distance to substrate: 40mm Working pressure $p_{O_{2}}:0.5$–3.6Pa Target: ${\mathrm{VO}}_{2}$	KrF excimer Frequency: 10Hz Fluence: – Deposition time: –
	Sapphire [79]	Substrate temperature: 600°C Working pressure $p_{O_{2}}:2.6$–4Pa Target: $V_{2}O_{3}$	ArF excimer Frequency: – Fluence: 2.0–$3.0\;J\cdot {\mathrm{cm}}^{-2}$ Deposition time: –
	Si/${\mathrm{SiO}}_{2}$ [80]	Substrate temperature: 600°C Distance to substrate: 35mm Working pressure $p_{O_{2}}:\;∼6.67\;\mathrm{Pa}$ Target: $V_{2}O_{5}$	KrF excimer Frequency: 10Hz Laser energy: 200mJ Deposition time: –
	Sapphire [81]	Substrate temperature: 600°C Distance to substrate: 90mm Working pressure $p_{O_{2}}:\;∼1.33\;\mathrm{Pa}$ Target: $V_{2}O_{5}$ Flow $F_{O_{2}}:10\;\mathrm{sccm}$	KrF excimer Frequency: – Fluence: – Deposition time: –
NiO	${\mathrm{SnO}}_{2}$:F (FTO)-coated glass [82]	Substrate temperature: RT–300°C Distance to substrate: 40–50mm Working pressure $p_{O_{2}}:0.1$–10Pa Target: NiO	KrF excimer Frequency: 5Hz Fluence: 1.0–$2.0\;J\cdot {\mathrm{cm}}^{-2}$ Deposition time: 30–60min
	${\mathrm{SnO}}_{2}$:F (FTO)-coated glass [83]	Substrate temperature: RT–300°C Distance to substrate: 55mm Working pressure $p_{O_{2}}:45\;\mathrm{Pa}$ Target: Ni-metal	KrF excimer Frequency: 20Hz Fluence: $2.0\;J\cdot {\mathrm{cm}}^{-2}$ Deposition time: –
	ITO-coated glass [84]	Substrate temperature: RT–400°C Working pressure $p_{O_{2}}:0.01$–0.016Pa Target: NiO Flow $F_{O_{2}}:10\;\mathrm{sccm}$	KrF excimer Frequency: 5–50Hz Laser energy: 125mJ Deposition time: –
${\mathrm{WO}}_{3}$	Si(100) [85]	Substrate temperature: 300–400°C Distance to substrate: 75mm Working pressure $p_{O_{2}}:2.67\;\mathrm{Pa}$ Target: ${\mathrm{WO}}_{3}$	ArF excimer Frequency: 10Hz Fluence: $1.5\;J\cdot {\mathrm{cm}}^{-2}$ Deposition time: 30min
	${\mathrm{SrTiO}}_{3}$ [86]	Substrate temperature: 300–800°C Working pressure $p_{O_{2}}:1$–6Pa Target: ${\mathrm{WO}}_{3}$	KrF excimer Frequency: 3Hz Fluence: 0.6–$1.2\;J\cdot {\mathrm{cm}}^{-2}$ Deposition time: –
${\mathrm{TiO}}_{2}$	ITO-coated glass, Si [87]	Substrate temperature: 25–700°C Distance to substrate: 80mm Working pressure $p_{O_{2}}:0.1\;\mathrm{Pa}$ Target: ${\mathrm{TiO}}_{2}$ (anatase and rutile)	Femtosecond laser (800nm,100fs) Frequency: 1kHz Fluence: $3.34\;J\cdot {\mathrm{cm}}^{-2}$ Deposition time: –
	Aluminum foil, Si(100) [88]	Substrate temperature: RT Distance to substrate: 30mm Without oxygen Target: ${\mathrm{TiO}}_{2}$ (anatase)	Nd:glass Frequency: 10Hz Laser energy: 2.8mJ Deposition time: 180min

**Table 13:** Magnetron sputtering deposition conditions for selected transition-metal oxide films.

TMO	Magnetron Sputtering Technique	Deposition Parameters	Substrate and Target Parameters	Ambient Conditions
${\mathrm{VO}}_{2}$	RF [99]	Sputtering power: 70W	Substrate: Corning glass Substrate temperature: 450°C Target: V	Working pressure: 1.8Pa
	RF [100]	Sputtering power: 350W	Substrates: Si(100), glass Substrate temperature: 500°C Distance to substrate: 90mm Target: V	Working pressure: 0.1–0.5Pa Flow rate $F_{O_{2}}:1.75\;\mathrm{sccm}$
	RF [101]	–	Substrates: ${\mathrm{SiO}}_{2}$/Si, c-plane sapphire Substrate temperature: 650°C Target: V	$p_{O_{2}}:56\;\mathrm{mPa}$ $p_{\mathrm{Ar}}:19.4\;\mathrm{mPa}$ Working pressure: 0.02Pa
	DC [102]	Sputtering power: 90–100W	Substrates: ${\mathrm{SiO}}_{2}$/Si, quartz sapphire Substrate temperature: 650°C Target: V	Working pressure: 0.02Pa Flow rate $F_{O_{2}}:1.3\;\mathrm{sccm}$ Flow rate $F_{\mathrm{Ar}}:100\;\mathrm{sccm}$
	Magnetron sputtering [103]	Power density on V-target: $1.1\;W\cdot {\mathrm{cm}}^{-2}$	Substrate: SLG Substrate temperature: 300–400°C Target: V	Working pressure: 0.02Pa Flow rate $F_{O_{2}}:1.8\;\mathrm{sccm}$ Flow rate $F_{\mathrm{Ar}}:40\;\mathrm{sccm}$
NiO	DC [104]	Sputtering power: 600W	Substrates: Al, KCl, glass Substrate temperature: RT Distance to substrate: 75mm Target: Ni	$p_{O_{2}}:0.15\;\mathrm{Pa}$ $p_{\mathrm{Ar}}:0.35\;\mathrm{Pa}$ Working pressure: 0.5Pa
	DC [47]	Sputtering power: 30–70W	Substrate: glass Substrate temperature: RT Distance to substrate: 75mm Target: Ni	$p_{O_{2}}/p_{\mathrm{Ar}}:2/1$ Working pressure: 1Pa
	RF [105]	Sputtering power: 90W	Substrate: SLG Substrate temperature: RT Target: NiO	Working pressure: 40mPa Flow rate $F_{\mathrm{Ar}}:6\;\mathrm{sccm}$
${\mathrm{WO}}_{3}$	DC [106]	Sputtering power: 200W	Substrate: ${\mathrm{CaF}}_{2}$ Substrate temperature: 280°C Target: W	$p_{O_{2}}/p_{\mathrm{Ar}}:0.43$ Working pressure: 1.33–4Pa
	RF [107]	Sputtering power: 120W	Substrate: ITO-coated glass Substrate temperature: RT Target: ${\mathrm{WO}}_{3}$	Working pressure: 0.4Pa Flow rate $F_{\mathrm{Ar}}:67\;\mathrm{sccm}$
${\mathrm{TiO}}_{2}$	DC [108]	Sputtering power: 0–50W	Substrate: Ti/FTO Substrate temperature: RT Distance to substrate: 115mm Target: Ti	$p_{O_{2}}:46\;\mathrm{mPa}$ Working pressure: 0.14Pa Flow rate $F_{\mathrm{Ar}}:10\;\mathrm{sccm}$ Flow rate $F_{O_{2}}:5\;\mathrm{sccm}$
	RF [109]	Sputtering power: 80–200W	Substrates: Si(100), glass Substrate temperature: RT–150°C Target: ${\mathrm{TiO}}_{2}$	Working pressure: 4–8Pa Flow rate $F_{O_{2}}:0$–30sccm
	RF [110]	Sputtering power: 500W	Substrate: p-Si(100) Substrate temperature: RT Distance to substrate: 44mm Target: ${\mathrm{TiO}}_{2}$	Working pressure: 0.6Pa Flow rate $F_{O_{2}}:0$–8.5sccm
	RF [111]	Sputtering power: 160W	Substrate: glass Substrate temperature: RT Target: ${\mathrm{TiO}}_{2}$	Working pressure: 0.6Pa Flow rate $F_{\mathrm{Ar}}:30\;\mathrm{sccm}$

**Table 14:** Sol–gel deposition conditions for selected transition-metal oxide films.

TMO	Substrate	PLD Parameters	Laser Type and Parameters
${\mathrm{VO}}_{2}$	Spin coating [117]	Substrate: Si Source of TM: $\mathrm{VO}{\left(\mathrm{acac}\right)}_{2}$ Sol: 100mM of $\mathrm{VO}{\left(\mathrm{acac}\right)}_{2}$ in ethanol	Drying at 150°C 1. humid $N_{2}$: 1.8–$2.3\;g_{H_{2}O}/{\mathrm{kg}}_{\mathrm{dry}-\mathrm{air}}$ (11–13% RH at 22–23°C) 2. dry air: 2.2–$3.2\;g_{H_{2}O}/{\mathrm{kg}}_{\mathrm{dry}-\mathrm{air}}$ (14–18% RH at 21–23°C) 3. ambient air: 5.7–$7.5\;g_{H_{2}O}/{\mathrm{kg}}_{\mathrm{dry}-\mathrm{air}}$ (34–45% RH at 22°C) 4. humid air: 12.5–$15.9\;g_{H_{2}O}/{\mathrm{kg}}_{\mathrm{dry}-\mathrm{air}}$ (85–90% RH at 20–23°C) Annealing at 550°C for 1h (5°C/min) under a 1.7L/min $F_{N_{2}}$ flux
	Spin coating [118]	Substrates: Si, quartz Source of TM: ammonium citrato-oxovanadate (IV) ${\left({\mathrm{NH}}_{4}\right)}_{4}{\left[V_{2}O_{2}{\left(C_{6}H_{4}O_{7}\right)}_{2}\right]}_{2}\cdot H_{2}O$ (CA-V(IV)) Sol: CA-V(IV) + ethanol + cetyltrimethylammonium bromide (CTAB) + distilled water, with varying molar ratio of CTAB to V	Annealing at 500°C for 1h in Ar atmosphere
	Spin coating [119]	Substrate: ${\mathrm{Al}}_{2}O_{3}\left(0001\right)$ Source of TM: vanadyl triisopropoxide $\mathrm{VO}{\left({\mathrm{OC}}_{3}H_{7}\right)}_{3}$ Sol: 50mL of $\mathrm{VO}{\left({\mathrm{OC}}_{3}H_{7}\right)}_{3}$, isopropanol and acetic acid with mass ratio of 1:20.1:3.2, respectively	Drying at 250°C for 3min Annealing at 370–520°C for 7h in $N_{2}$ with 500mL/min flow rate
NiO	Spin coating [120]	Substrate: glass Source of TM: nanocrystalline NiO powder Sol: nanocrystalline NiO powder + *m*-cresol	Drying at 100°C for 10min
	Spin coating [121]	Substrate: – Source of TM: nickel acetate tetrahydrate $\left[\mathrm{Ni}{\left({\mathrm{CH}}_{3}\mathrm{COO}\right)}_{2}\cdot 4H_{2}O\right]$ Sol: 2.54g of nickel acetate tetrahydrate + 100mL of 1M aqueous citric acid solution and 10mL of ethylene glycol	Drying at 250°C for 2h at atmospheric pressure Annealing at 800°C for 1h
	Spin & dip coating [122]	Substrates: ITO-coated glass, Corning glass Source of TM: Nickel(II) 2-ethylhexanoate ${\left[{\mathrm{CH}}_{3}{\left({\mathrm{CH}}_{2}\right)}_{3}\mathrm{CH}\left(C_{2}H_{5}\right){\mathrm{CO}}_{2}\right]}_{2}\mathrm{Ni}$ Sol: 42g of Nickel(II) 2-ethylhexanoate + 4mL isopropanol	Drying at RT in air for 10min Annealing at 350°C for 1h
${\mathrm{WO}}_{3}$	Spin coating [123]	Substrate: ITO-coated glass Source of TM: tungsten (VI) chloride (${\mathrm{WCl}}_{6}$) powder Sol: 1g of ${\mathrm{WCl}}_{6}$ 20mL of absolute ethanol 2mL of glacial acetic acid 2mL of $H_{2}O_{2}$	Drying at 100°C for 3min Annealing at 250°C
	Dip coating [124]	Substrate: glass Source of TM: $H_{2}{\mathrm{WO}}_{4}$ Sol: 6.72g of $H_{2}{\mathrm{WO}}_{4}$ + 10mL of $H_{2}O_{2}$	Drying at 100°C for 10min Air-annealing at 150–400°C for 1h (5°C/min)
	Sol–gel [125]	Substrates: FTO-coated glass, glass Source of TM: tungsten (VI) chloride (${\mathrm{WCl}}_{6}$) powder Sol: 1.1896g of ${\mathrm{WCl}}_{6}$ 10mL of $H_{2}O_{2}$ 30mL of 2-methoxyethanol	Drying at RT for 5min in air Air-annealing at 300°C for 1h
${\mathrm{TiO}}_{2}$	Dip coating [126]	Substrate: glass Source of TM: titanium tetra-isopropoxide (TTIP) $\mathrm{Ti}{\left[\mathrm{OCH}{\left({\mathrm{CH}}_{3}\right)}_{2}\right]}_{4}$ Sol: TTIP, 1-propanol, hydrochloric acid (HCl, 36%), and monoethanolamine	Drying at 100°C for 20min Preheated at 200°C for 10min Annealing at 500°C for 2h
	Dip coating [127]	Substrates: SLG, quartz Source of TM: titanium tetra-isopropoxide (TTIP) $\mathrm{Ti}{\left[\mathrm{OCH}{\left({\mathrm{CH}}_{3}\right)}_{2}\right]}_{4}$ Sol: TTIP, isopropanol ${\left({\mathrm{CH}}_{3}\right)}_{2}\mathrm{CHOH}$, glacial acetic acid ${\mathrm{CH}}_{3}\mathrm{COOH}$ and methanol ${\mathrm{CH}}_{3}\mathrm{OH}$	Drying at 100°C for 15min Annealing at 350–950°C for 1h
	Spin coating [128]	Substrate: glass Source of TM: nanocrystalline ${\mathrm{TiO}}_{2}$ powder Sol: 0.4g of nanocrystalline ${\mathrm{TiO}}_{2}$ powder 5mL of ethanol 5mL of diethylene glycol	Drying at 100°C for 10min Annealing at 400°C

**Table 15:** Summary of TMO thin film preparation technologies.

TMO Preparation Technology	Main Advantages	Main Limitations	Device Integration Aspects
Magnetron Sputtering	Scalable to large-area coatings; industry-compatible; good thickness and uniformity control	Requires optimization of oxygen partial pressure, stress, crystallinity, and post-annealing; reactive sputtering may suffer from target poisoning and process hysteresis; phase control can be challenging for multivalent oxides	Highly relevant for SW and multilayer coatings; compatible with glass substrates and industrial coating lines
PLD	Good stoichiometry transfer; suitable for complex and multicomponent oxides; precise control of deposition parameters, including oxygen pressure, laser fluence, and substrate temperature	Limited scalability; relatively small deposition area; possible formation of particulates/droplets; relatively high cost and low throughput	Excellent for model films and mechanism studies; less attractive for industrial large-area devices
Sol–gel	Low cost; simple equipment; compositional flexibility; potentially scalable to large-area coatings	Cracking, porosity, thickness non-uniformity, organic residues, low oxygen control, and the need for thermal treatment	Useful for low-cost coatings, but device reproducibility and long-term stability require careful control

**Table 16:** Dopants for tuning the MIT temperature and optical performance of VO_2_ films.

Dopant	$T_{\mathrm{lum}}$	$\Delta T_{\mathrm{sol}}$	$\Delta T_{c}$	Mechanism
$W^{6+}$ [151,152]	69.5%	3.4%	Decrease in ∼20–26°C/at.%	Increased free electron concentration
${\mathrm{Ti}}^{4+}$ [153,154]	53.0%	17.2%	Increase	Smaller ionic radius
${\mathrm{Nb}}^{5+}$ [155]	22–40%	–	Decrease in ∼2°C/at.%	Increased free electron concentration
${\mathrm{Mg}}^{2+}$ [156,157]	82.1%	4.8%	Decrease in ∼3°C/at.%	Increased free hole concentration
${\mathrm{Zr}}^{4+}$ [158]	60.4%	14.1%	Decrease in ∼0.4°C/at.%	Larger ionic radius
${\mathrm{Cr}}^{3+}$ [159]	4.0–23.8%	0.6–11.0%	Increase	Increased free hole concentration
${\mathrm{Sn}}^{4+}$ [160]	–	–	Increase in ∼1°C/at.%	Smaller ionic radius
${\mathrm{Eu}}^{3+}$ [161]	52–56%	5.3–6.7%	Decrease in ∼5°C/at.%	Larger ionic radius
${\mathrm{Mg}}^{2+}+W^{6+}$ [162]	81.3%	4.3%	Decrease	Increased free hole and electron concentrations
${\mathrm{Zr}}^{4+}+W^{6+}$ [158]	56.4%	12.3%	Decrease	Increased free electron concentration
${\mathrm{Mo}}^{6+}+W^{6+}$ [163]	–	–	Decrease	Increased free electron concentration

**Table 17:** AR materials for improving the luminous transmittance of ${\mathrm{VO}}_{2}$-based SWs.

AR Materials	*n* at λ = 550 nm	Design and Architecture
${\mathrm{SiO}}_{2}$ [166]	∼1.45	Top AR
${\mathrm{TiO}}_{2}$ [164]	2.40–2.60	Top and bottom AR, self-cleaning, photocatalyst
${\mathrm{SnO}}_{2}$ [167]	1.9–2.0	Top and bottom AR
ZnO [168]	1.9–2.0	Top and bottom AR
${\mathrm{Cr}}_{2}O_{3}$ [169]	2.40–2.60	Bottom AR + UV shielding
${\mathrm{WO}}_{3}$ [170]	2.00–2.10	Top and bottom AR
${\mathrm{ZrO}}_{2}$ [171]	2.05–2.15	Top and bottom AR
AlN [172]	2.00–2.10	Top and bottom AR

**Table 18:** Electrolytes used in TMO-based electrochromic windows.

Electrolyte Type	Typical Examples	Common Mobile Ions	Notes for TMO Electrochromic SW
Liquid/liquid-polymer electrolytes (LPEs) [180]	Propylene carbonate (PC), polyethylene glycol (PEG), polyethylene oxide (PEO) with salts such as LiClO_4_, LiI, LiTFSI, LiPF_6_	Mainly Li^+^	High ionic conductivity and easy processing, but limited by leakage, evaporation, bubble formation, and sealing/safety issues
Gel polymer electrolytes (GPEs)/quasi-solid electrolytes [180,181]	PVDF-HFP-based gels, PEGDA/PEO gels, UV-cured PMMA gels, ion gels	Mainly Li^+^	High ionic conductivity, better mechanical stability, and reduced leakage; common in WO_3_/NiO devices
Solid polymer electrolytes (SPEs) [180]	PMMA, gelatin, methyl-cellulose-based electrolytes, bio-based polymer electrolytes	Li^+^ or H^+^	Attractive for all-solid-state SWs because they eliminate leakage and simplify lamination/packaging, although conductivity can be lower than in liquid systems
Inorganic solid electrolytes [180,182]	Ta_2_O_5_, Li:Ta_2_O_5_, LiAlSiO_4_, phosphate glass, related oxide/ceramic ion conductors	H^+^, Li^+^, Na^+^ depending on composition	Important in all-solid-state SWs because they offer good film integration, durability, and device stability
Hybrid organic–inorganic polyelectrolytes [183]	Sol–gel-derived hybrid electrolytes, ORMOLYTE-type materials, siloxane-based hybrid polyelectrolytes	Mainly Li^+^	Designed to combine the mechanical robustness of inorganic networks with the processability and flexibility of polymers; reported in NiO/WO_3_ complementary devices

**Table 19:** Commercial electrochromic glazing products and reported optical performance.

Manufacturer	Product Name	Maximum Size (mm)	$T_{\mathrm{lum}}$ Range	$T_{\mathrm{sol}}$ Range
SageGlass www.sageglass.com (accessed date: 19 May 2026)	SageGlass Clear DGU	3095×1828	1%→60%	0.4%→33%
	SageGlass Blue DGU	3095×1828	0.5%→40%	0.3%→21%
View https://view.com/ (accessed date: 19 May 2026)	View Gen 4 DGU–Clear	1828×3048	1%→53%	0%→29%
	View Gen 4 DGU–Clear + SN68 low-e	1828×3048	1%→45%	0%→19%
	View Gen 4 Laminated DGU–clear/$0.060^{\prime \prime }$ PVB/clear	1828×3048	1%→52%	0%→27%
	View Gen 4 TGU–Clear ×2	1828×3048	1%→47%	0%→24%
	View Gen 4 TGU–Clear ×2 + SN68 low-e	1828×3048	1%→40%	0%→16%
Vitrum Glass Group https://www.vitrum.ca/ (accessed date: 19 May 2026)	Halio	3058×1508	2%→65%	–
	Halio Black	3058×1508	0.01%→52%	–
ConverLight https://converlight.com/ (accessed date: 19 May 2026)	ConverLight Dynamic 75 2G	1550×4400	36%→67%	21%→41%
	ConverLight Dynamic 75 3G	1550×4400	33%→61%	17%→34%
	ConverLight Dynamic 65 3G	1550×4400	14%→56%	7%→31%
	ConverLight Dynamic 65 4G	1550×4400	13%→51%	6%→26%

## Data Availability

No new data were created or analyzed in this study. Data sharing is not applicable to this article.

## Abbreviations

The following abbreviations are used in this manuscript:

AACVD	Aerosol-assisted chemical vapor deposition
AFI	Antiferromagnetic insulating
ALD	Atomic layer deposition
AR	Antireflection
AZO	Aluminum-doped zinc oxide
CE	Coloration efficiency
CT	Charge-transfer
CVD	Chemical vapor deposition
DFT	Density functional theory
DGU	Double glazing unit
ECW	Electrochromic window
HiPIMS	High-power impulse magnetron sputtering
HRTEM	High-resolution transmission electron microscopy
IGU	Insulating glass unit
IR	Infrared
ITO	Indium tin oxide
LCST	Lower critical solution temperature
LPCVD	Low-pressure chemical vapor deposition
LPEs	Liquid/liquid-polymer electrolytes
MBE	Molecular beam epitaxy
MIR	Mid-infrared
MIT	Metal–insulator transition
MOX	Metal oxide
NIR	Near-infrared
PC	Propylene carbonate
PLD	Pulsed laser deposition
RFMS	Radio-frequency magnetron sputtering
RH	Relative humidity
SEM	Scanning electron microscopy
SHGC	Solar heat gain coefficient
SLG	Soda-lime glass
SPEs	Solid polymer electrolytes
SW	Smart window
SWs	Smart windows
TEM	Transmission electron microscopy
TGU	Triple glazing unit
TMM	Transfer matrix method
TMO	Transition metal oxide
TMOs	Transition metal oxides
UV	Ultraviolet
VIS	Visible
YSZ	Yttria-stabilized zirconia
